# Rare pathogenic structural variants show potential to enhance prostate cancer germline testing for African men

**DOI:** 10.1038/s41467-025-57312-9

**Published:** 2025-03-10

**Authors:** Tingting Gong, Jue Jiang, Korawich Uthayopas, M. S. Riana Bornman, Kazzem Gheybi, Phillip D. Stricker, Joachim Weischenfeldt, Shingai B. A. Mutambirwa, Weerachai Jaratlerdsiri, Vanessa M. Hayes

**Affiliations:** 1 Ancestry and Health Genomics Laboratory, Charles Perkins Centre, School of Medical Sciences, Faculty of Medicine and Health, University of Sydney, Camperdown, NSW 2050 Australia; 2 Human Phenome Institute, Fudan University, Shanghai, China; 3 School of Health Systems and Public Health, University of Pretoria, Pretoria, South Africa; 4 St Vincent’s Prostate Cancer Research Centre, Sydney, NSW Australia; 5 Finsen Laboratory, Rigshospitalet, DK-2200 Copenhagen, Denmark; 6 Biotech Research & Innovation Centre, University of Copenhagen, DK-2200 Copenhagen, Denmark; 7 Department of Urology, Sefako Makgatho Health Science University, Dr George Mukhari Academic Hospital, Medunsa, Ga-Rankuwa, South Africa; 8 Manchester Cancer Research Centre, University of Manchester, Manchester, M20 4GJ UK

**Keywords:** Prostate cancer, Cancer screening, Cancer genomics, Cancer genetics

## Abstract

Prostate cancer (PCa) is highly heritable, with men of African ancestry at greatest risk and associated lethality. Lack of representation in genomic data means germline testing guidelines exclude for Africans. Established that structural variations (SVs) are major contributors to human disease and prostate tumourigenesis, their role is under-appreciated in familial and therapeutic testing. Utilising clinico-methodologically matched deep-sequenced whole-genome data for 113 African *versus* 57 European PCa patients, we interrogate 42,966 high-quality germline SVs using a best-fit pathogenicity prediction workflow. We identify 15 potentially pathogenic SVs representing 12.4% African and 7.0% European patients, of which 72% and 86% met germline testing standard-of-care recommendations, respectively. Notable African-specific loss-of-function gene candidates include DNA damage repair *MLH1* and *BARD1* and tumour suppressors *FOXP1, WASF1* and *RB1*. Representing only a fraction of the vast African diaspora, this study raises considerations with respect to the contribution of kilo-to-mega-base rare variants to PCa pathogenicity and African-associated disparity.

## Abstract (web summary)

In prostate cancer, the contribution of germline structural variants (SVs) remains under-appreciated, especially in patients with African ancestry. Here, the authors identify potentially pathogenic germline SVs and their possible functional and clinical impact in whole-genome sequencing data from African men with prostate cancer.

## Introduction

Prostate cancer (PCa) is a significant global health burden and a leading cause of male-associated cancer deaths^1^. With one of the highest heritability rates (estimated 58%), PCa risk shows a great degree of variability^2^, particularly when considering a man’s ancestral heritage. In the United States, Black men are at greatest risk for aggressive disease presentation^3^ and, depending on age at diagnosis over double to triple (< 65 years) the risk for PCa-associated mortality than White Americans^4,5^. Contributed by a complex interaction of socioeconomic factors and genetics^6^, inherited risk includes a combination of both common (low-risk with combined genetic risk scores) and rare (high-risk or pathogenic) germline variants^7,8^. Revolutionised through the advancement of precision oncology, most notably the approval of the poly-(ADP ribose) polymerase (PARP) inhibitors Olaparib^9^ and Rucaparib^10^ for the treatment of metastatic castrate-resistant PCa for patients harbouring rare pathogenic variants in specified DNA repair genes^11^, has increased the value for germline testing. Furthermore, the National Comprehensive Cancer Network (NCCN) recommends germline testing for all men with metastatic, recurrent or high-risk localised PCa, regardless of family history^12^. Although a significant risk factor for aggressive disease, no consensus could be reached for men of African ancestry^13^, while a recent review further highlighted the knowledge gap^14^.

The lack of consensus for PCa germline testing in Black men is directly attributed to a lack of available data, compounded by a lack of African-relevant genomic data that captures the true extent of elevated genetic diversity. While consensus has yet to be reached for minority inclusion in the benefits of recent breakthroughs in PCa precision oncology, contradictory studies suggest that Black American patients harbour more^15^ and conversely less actionable pathogenic variants than White Americans^16^. The picture is no different for Africa, although more recently PCa genomics has reached the continent, with the inclusion of whole exome (*n* = 45 Nigerian)^17^ and whole genome sequencing studies (*n* = 113 Black South Africans)^18^. Although preliminary, notable differences within Africa are emerging. For example, an elevated frequency of *BRCA1* germline mutations reported for Nigerian patients, reflecting African American data^17,19^, is lacking in Southern African cases^20^. In addition, we have recently reflected on the lack of the West African exclusive and functionally relevant common PCa susceptibility variants *CHEK2* p.Ile448Ser (rs17886163) and *HOXB13* p.Ter285Lys (rs77179853) in Southern Africa^21,22^. Reporting a 2.1-fold age-adjusted increase in aggressive PCa presentation in Black South African *versus* Black American men^23^, through deep sequenced interrogation for the 20 most common genes included in PCa germline testing panels using NCCN inclusion criteria (Gleason score ≥ 8), we observed a prevalence for rare pathogenic variants of 5.6%^20^, comparable with a single East African study (5.7%)^24^ and almost half that reported for non-Africans (11.8%)^25^. These studies highlight the need for developing African-relevant PCa germline testing panels through African inclusion in genome profiling.

Again, it is well established that Structural Variations (SVs) play a critical role in prostate tumour progression with prognostic and therapeutic potential^26,27^, including tumours derived from men of African ancestry^18,28^. Yet, irrespective of patient ancestry, little is known with regard to the contribution of germline potentially pathogenic rare SVs. Typically, greater than 50 bases in length, SVs encompassing large deletions (DEL), duplications (DUP), insertions (INS), inversions (INV) and translocations (TRA), are overlooked and/or difficult to resolve using current germline genetic testing assays. While it is well established that SVs play a critical role in diagnostic screening for inherited genetic diseases^29^, more recently, long-read sequencing has been used to identify potential pathogenic SVs in hereditary cancer syndromes^30^ and known breast cancer susceptibility genes^31^, however, the impact of rare pathogenic SVs on PCa predisposition, and in turn targeted treatment, remains unknown.

Here, expanding on our earlier work^18,20,28^, including deep sequenced germline genomes for 113 African (Black South African) and 57 European (4 South African, 53 Australian) PCa patients, through high-quality SV calling and genotyping, comprehensive gene annotation and best-fit pathogenicity prediction, we interrogate for rare potentially pathogenic SVs (PP-SVs). While agreeably a small study size, this resource is not only unique for the African continent, importantly it provides clinically and technically matched non-African data for direct comparative analyses, while our whole genome approach increases sensitivity for SV detection. We provide computational and case-associated expression evidence for PP-SV contribution to aggressive PCa presentation and associated ancestral disparities, including unknown to PCa, ancestry-specific germline testing gene candidates. Our study reveals the added value for whole-genome germline SV interrogation and African inclusion to provide important insights into optimising PCa germline testing for global impact.

## Results

### NCCN high-risk characterisation for ancestrally assigned PCa patients

Clinically and technically matched whole genome sequenced germline data (mean coverage 45.9X; range 30.2–97.6X) was derived from whole blood from 170 PCa patients, ancestrally classified previously using 7,472,833 genome-wide SNVs and population substructure analysis^18^. In brief, 113 Black South African patients presented with an African ancestral genetic fraction of > 85%, while the 57 White patients presented with European ancestral genetic fractions of > 90% (4 South African, 52 Australian) and 73.7% European and 26.3% Asian substructure (1 Australian) (Supplementary Table 1). Importantly, although the mean age was 5 years younger at presentation or surgery, a greater number of European (86%; 49/57) over African patients (72%; 81/113) met current NCCN guidelines for germline testing based on International Society of Urological Pathology (ISUP) Group Grading defined as high-risk localised PCa (ISUP 4/5 or Gleason score $\ge$ 8). Notably, we have previously provided evidence for the extension of these criteria for Black South African men to include ISUP 3, which would expand our cohort of high-risk Black men to 82% (93/113)^20^. While Black South Africans present with significantly elevated median and range of prostate-specific antigen (PSA) levels (median 244 ng/mL *versus* 9.4), as previously presented^18,23^, still the study was biased towards over-representation of NCCN guidelines for PSA-inclusive high-risk PCa for the European (70.2%; 40/57) over African patients (65/113; 57.5%).

### Genome-wide gene-disrupting SV discovery

**Fig. 1:**
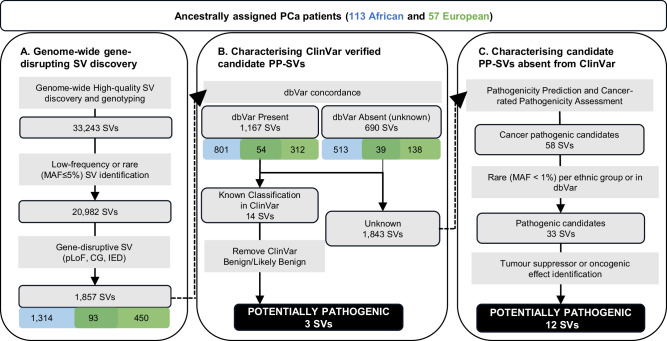
Workflow of PCa potentially pathogenic SV (PP-SV) identification. Including genome-wide gene-disrupting SV discovery (**A**) identifying known potential pathogenic SVs (**B**), while further characterising SVs of unknown significance (**C**). The detailed criteria to predict the potential pathogenicity were shown in Supplementary Table 4. The identification of tumour suppressor or oncogenic effect for disrupted genes by pathogenic candidates and the related literature were shown in Supplementary Table 7.

In this study, we identified and genotyped 42,966 high-quality germline SVs. We found a median of 9206 SVs (range: 8891– 9708) per African genome, which is significantly higher than the median of 7490 (range: 7309–8050) per European genome (*p*-value = 1.1e-26 by Wilcoxon test). In total, we identified 38,668 African-derived SVs (18,674 private) and 24,292 European-derived SVs (4298 private) (Supplementary Table 2). Including only high-quality genotype calls for allele frequency (AF) estimation left a total of 33,243 high-confidence SVs. Excluding common SVs, defined as minor allele frequency (MAF) > 5%, a total of 20,982 rare (MAF $\le \,$1%) and low-frequency (MAF = 1 to 5%) SVs remained across the ancestries for further annotation (Fig. 1).

Further interrogation for gene regions overlapping, we identified 1857 gene-disruptive SVs, including 1752 potential Loss-of-Function (pLoF), 52 Copy Gain (CG) and 53 Intragenic Exon DUP (IED) (detailed in Methods). Notably, pLoF, CG and IED SVs can have a functional impact on genes through either gene inactivation or increased dosage effect^32^. Conversely, there is no clear or direct coding effect by SVs with other gene impact types, which included in our study 109 partial gene DUP, 22 partial exons DUP, 48 whole-gene INV, 343 promoter SVs, 9431 intronic SVs and 258 enhancer SVs. As such, the latter SVs were not discussed further. In total, we identified 1857 (MAF $\le \,$5%) gene-disruptive SVs of which 1407 are African-relevant, including 93% (1314) African-private, and 543 European-relevant, including 83% (450) European-private (Supplementary Table 2). There were 93 SVs (5%) shared by both African and European PCa patients. The 1857 gene-disruptive SVs (1050 rare in both African and European) underwent further downstream interrogation for potential clinical relevance. Of the 1857 gene-disruptive SVs, 1167 were previously reported in the dbVar database of SVs, while 690 were absent, of which 513 (74%) are uniquely African (Fig. 1).

### Characterising ClinVar verified candidate potentially pathogenic SVs

Of the 1167 dbVar-reported gene-disruptive SVs, 14 (1.2%) were recorded in ClinVar, with three reported as ‘pathogenic’ or ‘likely pathogenic’ based on functional prediction consensus. One 2958 bp likely pathogenic DEL results in loss of exon 7 in *OCA2* (Supplementary Fig. 1), a 5064 bp pathogenic DEL leads to exon 5–7 loss in *PIGN* (Supplementary Fig. 2), while a 235 bp likely pathogenic DUP duplicates exon 3 of *SLC3A1* (Supplementary Fig. 3). The *OCA2* and *PIGN* DELs were identified in a single African patient each, while the *SLC3A1* DUP presented in two African patients (Table 1).

**Table 1:** Candidate potentially pathogenic (PP) SVs identified in 170 PCa patients

Genes	Gene impact type^1^	1stChr	pos1	2ndChr	pos2	SV type	ClinVar / dbVar concordance	MAF African (this study)	MAF African (control)^2^	MAF European (this study)	MAF African (dbVar)^3^	MAF European (dbVar)^3^
**Potentially Pathogenic SV (PP-SV)**												
*SLC3A1*	IED	chr2	44281377	chr2	44281612	DUP	L-pathogenic	0.01 ^4^	0	0	0.0075	1.3e-04
*OCA2*	pLoF	chr15	28017719	chr15	28020677	DEL	L-pathogenic	0.004	0.02	0	0.0015	0.001
*PIGN*	pLoF	chr18	62152637	chr18	62157701	DEL	Pathogenic	0.004	0	0	0.0013	1.3e-04
*SLC7A2*	pLoF	chr8	17418976	chr8	17544122	DEL	In dbVar	0.009	0	0	0.003	0
*DNAJC15*	pLoF	chr13	43078470	chr13	43079390	DEL	In dbVar	0	0	0.009	0	1.0e-04
*BCL2L11*	pLoF	chr2	111122626	chr2	111125901	DEL	This study	0.005	0	0	NA	NA
*BARD1*	pLoF	chr2	214768022	chr2	214772899	DEL	This study	0.005	0	0	NA	NA
*COL4A2/ COL4A1*	CG	chr13	110294204	chr13	110633815	DUP	In dbVar	0.005	0	0	1.3e-04	6.3e-06
*SLC2A5*	IED	chr1	9045605	chr1	9049441	DUP	In dbVar	0	0	0.009	7.3e-04	0.002
*FOXP1*	pLoF	chr3	71097066	chr3	74525618	INV	This study	0.009	0	0	NA	NA
*WASF1*	pLoF	chr6	108167886	chr6	110172775	INV	In dbVar	0.004	0	0	9.6e-05	0
*MLH1*	pLoF	chr3	37000362	chr3	39352689	INV	In dbVar	0.004	0	0	4e-04	6.4e-06
*RB1*	pLoF	chr13	48466588	chr13	48473911	INV	In dbVar	0.004	0	0	1.8e-04	1.3e-05
*CTNNA1*	pLoF	chr5	138903881	chr19	21614900	TRA	This study	0	0	0.009	NA	NA
*AK8-DST*	pLoF	chr9	132876361	chr6	56896165	TRA	This study	0	0	0.009	NA	NA
**PP-SV candidates classified as ‘cautionary’**												
*LTBP1/BIRC6*	CG	chr2	32403832	chr2	33107415	DUP	In dbVar	0	0	0.009	1.0e-04	0.0018
*PHC3-PRKACA*	pLoF	chr3	170090742	chr19	14110142	TRA	This study	0.004	0	0	NA	NA
*KCTD3-DST*	pLoF	chr1	215567414	chr6	56652607	TRA	This study	0.009	0	0	NA	NA
*PKHD1*	pLoF	chr6	51981375	chr15	30874073	TRA	This study	0.009	0	0	NA	NA

*CG* copy gain, chr chromosome, *DEL* deletion, *DUP* duplication, *IED* intragenic exon duplication, *INV* inversion, L-pathogenic Likely pathogenic, *MAF* minor allele frequency, *pLoF* potentially loss-of-function, pos position, *TRA* translocation. Note, all gene names are in italics.

^1^Gene impact type based on gene annotation.

^2^Population-matched Southern African non-cancer control cohort (*n* = 49).

^3^The ancestry-related MAF in dbVar were based on gnomAD^32^ or TOPMed^42^ SV study. The details of all dbVar studies (dbVar study name and ID) and reported allele frequencies were shown in Supplementary Data 1.

^4^Presenting at low-frequency rather than rare variants within the ancestrally-defined patient cohort.

Although pathogenic in ClinVar, none have been associated with cancer phenotypes and include rather oculocutaneous albinism, multiple congenital anomalies-hypotonia-seizures syndrome and cystinuria, respectively. As such, we searched the literature for plausibility with further ascertainment derived from normal prostate and tumour tissue data sets using GENT2^33^. Reported to be downregulated in numerous cancer types (all-type *p*-value < 0.001, GENT2 T-test), although not significant for PCa, pLoF deletion of the pigmentation gene *OCA2* has been linked not only to Prader-Willi syndrome, but also Prader-Willi associated malignancies^34^, and melanoma^35^, with recent studies linking melanoma with increased PCa risk^36^. Highly expressed in normal prostate tissue with significant upregulation in tumour tissue (*p*-value < 0.001, GENT2 T-test), *PIGN* functions as a cancer chromosomal instability suppressor gene^37,38^. Although at lower levels, *SLC3A1* is also upregulated in PCa (*p*-value < 0.001, GENT2 T-test), with overexpression in breast cancer associated with tumourigenesis^39^. These observations, taken together, provide the rational for characterising the pLoF *OCA2* and *PIGN* DELs and *SLC3A1* IED as potentially pathogenic SVs (PP-SVs). Notably, all three SVs are reported as rare (irrespective of ancestry) in multiple population-wide studies including gnomAD SV^32^, 1000 genomes Project (1KGP)^40,41^ and TOPMed SV^42^ (Supplementary Data 1). However, the *OCA2* PP-SV was observed at a low frequency (1% <MAF $\le \,$5%) in our Southern African population-matched control data including whole genomes derived from 49 younger aged ( < 45 years) largely female (41, age range: 18–44) over male participants (8, age range: 18–39). Further gene interrogation showed an additional pLoF TRA on *OCA2*, resulting in *DNAH9*-*OCA2* gene fusion (Supplementary Table 3).

### Characterising candidate potentially pathogenic SVs absent from ClinVar

Among 1843 SVs with unknown classification in ClinVar or absent from dbVar, we predicted their potential pathogenicity based on four SV impact prediction tools, including StrVCTVRE^43^, CADD-SV^44^, POSTRE^45^ and PhenoSV^46^. The number of scored SVs by four tools and their types were shown in Supplementary Fig. 4 and Supplementary Table 4. Candidate SVs were required to meet two of the following criteria: StrVCTVRE score $\ge \,$0.37, CADD-SV score $\ge$ 10, POSTRE score $\ge \,$0.8 and/or PhenoSV score $\ge \,$0.5 (Supplementary Table 5 and Methods). Based on this criterion, all three ClinVar identified pathogenic or likely pathogenic SVs and the single SV of uncertain significance were successfully annotated as pathogenic candidates, while conversely, our workflow excluded for all 10 ClinVar characterised benign SVs (Supplementary Table 6). Using our criteria, 291 SVs were defined as PP-SV candidates (107 DELs, 16 DUPs, 11 INVs and 157 TRAs) disrupting 419 genes. In total, 190 candidate SVs were private to African and 88 to European patients, with 13 shared between the ancestries (Supplementary Table 5).

To further define cancer-related pathogenic potential, we assessed for the presence of disrupted genes by PP-SV candidates in gene sets derived from the Human Molecular Signature Database (MSigDB) oncogenic signature and hallmark gene sets^47^ and COSMIC Cancer Gene Census (COSMIC CGC) cancer driver genes^48^. Requiring disrupted genes in two of the three cancer gene sets, 58 SVs were defined as cancer-related PP-SV candidates, including 20 DELs, 3 DUPs, 6 INVs and 29 TRAs, disrupting 56 genes. Of the 58 candidates, 23 of them were identified with MAF between 1% to 5% in either African or European patients, leaving 35 rare PP-SV candidates for further consideration, of which 16 have been reported in dbVar. Two dbVar SVs, including TRA disrupting gene *NBEA* and *POLR2C* DEL, were reported at low frequencies (AF = 0.03 and 0.01, respectively) (Supplementary Data 1) and were therefore excluded from further analysis. Using our criteria, 33 rare cancer-related PP-SV candidates were identified (Supplementary Data 2 and Fig. 1), including 15 DELs, 3 DUPs (1 IED and 2 CGs), 5 INVs and 10 TRAs.

**Fig. 2:**
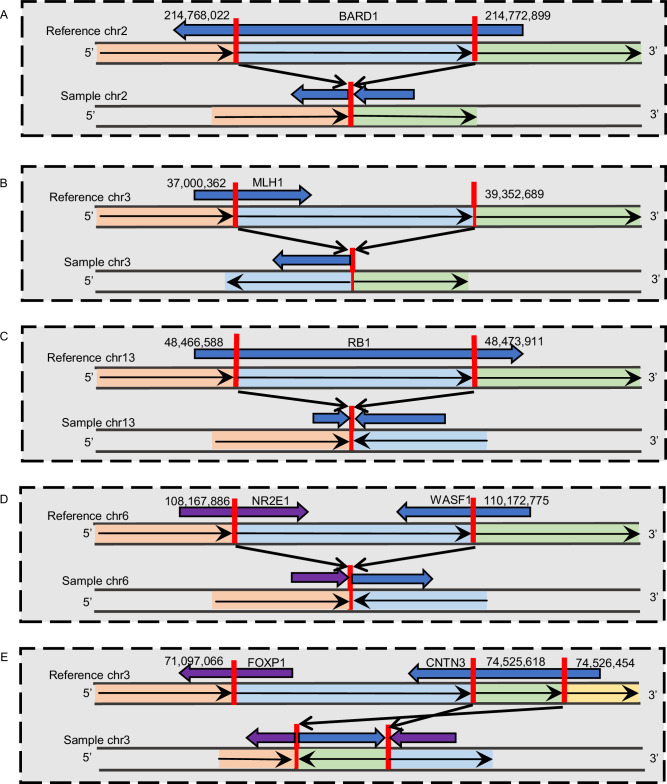
African-specific PP-SVs disrupting well-known pathogenic cancer genes and/or PCa tumour suppressor genes, including DNA damage response genes. **A** 4877 base pLoF deletion on DNA damage repair gene *BARD1*. **B** pLoF INV impacting PCa DNA mismatch repair gene *MLH1*. **C** pLoF INV impacting PCa tumour suppressor *RB1*. **D** pLoF INV impacting PCa tumour suppressor gene *WASF*1. **E** pLoF INV impacting PCa tumour suppressor gene *FOXP1*. More details of SV region and/or breakpoints on impacted genes and visual inspection of sequencing reads using Integrative Genomic Viewer^51^ are shown in Supplementary Figs. 8, 12, 13, 14 and 15, respectively.

Of the 15 pLoF DELs, 11 were excluded as PP-SVs, with impacting genes showing oncogenic behaviour in multiple cancer types or no strong evidence for their tumour suppressor effects (Supplementary Table 7). Conversely, four pLoF DELs were defined as PP-SVs, impacting known tumour suppressors or established DNA damage repair genes (Supplementary Table 7). Two of them are known to dbVar, including a *SLC7A2* 125,146 bp DEL identified in two African (Supplementary Fig. 5) and a *DNAJC15* 920 bp DEL in a European patient (Supplementary Fig. 6). Another two identified PP-SVs are unknown pLoF DELs, which identified in a single African patient each, including a *BCL2L11* 3,275 bp (Supplementary Fig. 7) and DNA damage repair gene *BARD1* 4877 bp DEL (Fig. 2A and Supplementary Fig. 8).

Of the two dbVar whole-gene DUPs, the *COL4A2* 339,611 bp CG, with breakpoints disrupting *COL4A1* and *NAXD*, observed in a single African patient is defined as a PP-SV (Supplementary Fig. 9), as *COL4A2* indicating oncogenic behaviour in gastric and breast cancers (Supplementary Table 7). In contrast, the *TTC27* 703,583 bp DUP observed in a single European patient is afforded ‘cautionary’ PP-SV status (Supplementary Fig. 10). Although *TTC27* is absent in three cancer gene databases, the breakpoints disrupt MSigDB and COSMIC CGC genes *BIRC6* and *LTBP1*, resulting in a *LTBP1*-*BIRC6* gene fusion of unclear effect. Observed in a single European patient, a 3836 base DUP directly impacts exon 4 of *SLC2A5* (Supplementary Fig. 11), which downregulated in PCa (*p*-value < 0.001, GENT2 T-test) and has been identified as an oncogenic behaviour (Supplementary Table 7), therefore allocated PP-SV status.

Of the five pLoF INVs, those impacting *MLH1*, *RB1* and *WASF1* are in dbVar, while *FOXP1* and *NSD3* INVs are unknown. As *NSD3* has been identified as oncogenic in multiple cancers, the associated INV is classified here as unlikely pathogenic, with all remaining pLoF INVs classified as PP-SVs, as they disrupting known to PCa and Lynch Syndrome predisposing DNA mismatch repair gene *MLH1* and PCa tumour suppressor genes *RB1*, *WASF1*, and *FOXP1* (Supplementary Table 7). Identified in a single African patient each (Supplementary Fig. 12–14), the three dbVar INVs were reported as rare by the recent TOPMed SV study^42^, in which *WASF1* INV was also identified as African-specific (Table 1 and Supplementary Data 1). The unknown INV impacting *FOXP1* was identified in two African patients (Fig. 2E and Supplementary Fig. 15).

Of the 10 pLoF TRAs, five impacting genes of *GRM8*, *WDR43*, *NPM1*, *NUSAP1* and *MECOM* with oncogenic properties (Supplementary Table 7), therefore, are classified as unlikely pathogenic. *PKHD1* TRA identified in two African patients received a ‘cautionary’ PP-SV classification, as identified as potential oncogenic in colon cancer, while potential tumour suppressor in colorectal cancer (Supplementary Table 7). As *CTNNA1* was known to have tumour suppressor behaviour across multiple tumour types (Supplementary Table 7), here we classify the European-specific pLoF *CTNNA1* TRA as a PP-SV (Supplementary Fig. 16). The remaining pLoF TRAs result in *PHC3-PRKACA* (1 African patient), *KCTD3-DST* (2 African patients) and *AK8-DST* (1 European patient, Supplementary Fig. 17) as current unknown gene fusions. *PHC3-PRKACA* was classified as ‘cautionary’ PP-SV, as *PHC3* showed a potential cancer suppressor effect in PCa, while *PRKACA* appears to portray oncogenic behaviour (Supplementary Table 7). Although unknown to PCa, both *DST* and *AK8* have demonstrated tumour suppressor behaviour, conversely, *KCTD3* with an unclear role in cancer (Supplementary Table 7). Here we classify *AK8-DST* as a PP-SV, while *KCTD3-DST* is assigned ‘cautionary’ PP-SV status.

### PP-SVs associated with clinical characters, expression and tumour features of causality

The clinicopathological features of the study cohort has been previously described^18,28^. In brief, African patients show a 5-year greater mean age and 25-fold greater PSA level at diagnosis compared to European patients (Supplementary Table 1). Based on our previous observations^20^, high-risk or aggressive PCa were defined as ISUP GG${{{\boldsymbol{\ge }}}}$3 and conversely, low-risk disease presentation as ISUP GG${{{\boldsymbol{ < }}}}$3. Biased towards aggressive disease presentation (82% African, 86.0% European), it was notable that all four patients with a pathogenic or likely pathogenic SVs presented with the aggressive disease at diagnosis, 92.9% (13/14) of PP-SV and 83.3% (5/6) cautionary PP-SV presenting patients (Table 2). We further concur that all SVs are likely germline as their variant allele frequencies (VAFs) are as expected for heterozygous inheritance (average of 41.8%; range 30% to 51%, Supplementary Table 8). Due to the lack of available Southern African population-matched non-cancerous data, here we further interrogated 49 younger-aged non-cancerous Southern Africans for potentially pathogenic SVs within the 19 PP-SV-defined candidate genes. Besides the previously discussed *OCA2* ClinVar defined “likely pathogenic” SV, no PP-SVs were identified within this cohort (Table 1). In turn, additional non-matched pLoF SVs were identified impacting *OCA2*, *BARD1* and *SLC2A2* (Supplementary Table 3).

**Table 2:** Clinicopathological features, variant allele frequencies (VAFs), associated somatic loss of heterozygosity (LOH) and/or second gene hit of prostate cancer (PCa) patients by ancestry presenting with potentially pathogenic (PP) SVs and cautionary PP-SVs as defined by this study

Gene name	Pathogenicity	SV (impact) type	Patient No.	Patient ID^2^	Expression^1^	VAF^3^	Ancestry	Age	PSA	ISUP GG	Tumour LOH^4^	Tumour 2^nd^ hit^5^
*SLC3A1*	PP-SV (LP)	DUP (IED)	1	N0001	ND	0.33	African	75	22.9	4	LOH-neg	No
–	–		2	SMU094	ND	0.30	African	64	15	4	LOH-CNG	CNL
*OCA2*	PP-SV (LP)	DEL (pLoF)	1	N0059	ND	0.45	African	79	153	5	LOH-neg	CNG
*PIGN*	PP-SV (P)	DEL (pLoF)	1	SMU083	ND	0.51	African	86	40.5	3	LOH-CNL	No
*SLC7A2*	PP-SV	DEL (pLoF)	1	UP2035	ND	0.48	African	70	680	5	LOH-neg	CNL
–	–		2	KAL0054	ND	0.49	African	64	42.9	5	LOH-CNG	No
*DNAJC15*	PP-SV	DEL (pLoF)	1	17135	ND	0.48	European	63	7.8	5	LOH-neg	No
*BCL2L11*	PP-SV	DEL (pLoF)	1	KAL0101	Low	0.45	African	71	32.3	5	LOH-CNN	No
*BARD1*	PP-SV	DEL (pLoF)	1	N0073	ND	0.49	African	62	unk	unk	LOH-CNN	No
*COL4A2/COL4A1*	PP-SV	DUP (CG)	1	UP2039	High/NE	0.35	African	71	319	4	LOH-neg	No
*SLC2A5*	PP-SV	DUP (IED)	1	11099	ND	0.33	European	70	9.9	5	LOH-neg	No
*FOXP1*	PP-SV	INV (pLoF)	1	UP2101	Low	0.41	African	57	75	5	LOH-neg	CNG
–	–	–	2	N0084	ND	0.41	African	65	591	4	LOH-neg	No
*WASF1*	PP-SV	INV (pLoF)	1	N0048	ND	0.32	African	70	83.3	5	LOH-neg	CNG
*MLH1*	PP-SV	INV (pLoF)	1	SMU080	Low	0.43	African	64	23.3	4	LOH-neg	No
*RB1*	PP-SV	INV (pLoF)	1	SMU064	ND	0.37	African	70	13.7	3	LOH-neg	No
*CTNNA1*	PP-SV	TRA (pLoF)	1	13179	ND	0.50	European	59	8.4	5	LOH-neg	No
*AK8-DST*	PP-SV	TRA (pLoF)	1	11452	ND	0.47	European	67	11	1	LOH-neg	No
*LTBP1/BIRC6*	Cautionary PP-SV	DUP (CG)	1	5287	ND	0.39	European	54	4.3	5	LOH-neg	CNG
*PHC3-PRKACA*	Cautionary PP-SV	TRA (pLoF)	1	SMU061	Avg./Avg.	0.49	African	65	12.1	3	LOH-neg	CNL
*KCTD3-DST*	Cautionary PP-SV	TRA (pLoF)	1	UP2039	ND	0.40	African	71	319	4	LOH-neg	CNL
–	–	–	2	SMU101	ND	0.42	African	70	4.3	3	LOH-neg	No
*PKHD1*	Cautionary PP-SV	TRA (pLoF)	1	N0056	ND	0.36	African	70	153	5	LOH-neg	CNG
			2	SMU196	ND	0.39	African	47	9.5	1	LOH-neg	No

*CG* copy gain, *CNG* copy-number gain, *CNL* copy-number loss, *CNN* copy-number neutral, *DEL* deletion, *DUP* duplication, *IED* intragenic exon duplication, *INV* inversion, *ISUP GG* International Society of Urological Pathology Group Grading, *TRA* translocation, *LOH* loss of heterozygosity, *LP* likely pathogenic, *ND* not determined, *NE* no expression, *neg* negative, *P* pathogenic, *pLoF* potentially loss-of-function, *SV* structural variant, *unk* unknown, *VAF* variant allele frequency. Note, all gene names are in italic; age is in years and PSA in ng/mL. Pathogenic (P) and Likely pathogenic (LP) are ClinVar defined.

^1^Gene expression derived from blood-matched RNAseq analysis. ^2^While no known family history of prostate, breast or ovarian cancer was reported for these patients, SMU080 reported a sister with cervical cancer and SMU061 a mother with stomach cancer. ^3^Variant allele frequency (Supplementary Table 8). ^4^Loss of heterozygosity status was inferred from TITAN. ^5^The details of the second somatic hit locations were shown in Supplementary Table 9.

In parallel, blood was available for 20 patients including five with a PP-SV allowing for further experimental expression analyses through RNA-sequencing interrogation. Consequently, the unknown pLoF PP-SVs impacting *BCL2L11* (KAL0101) and *FOXP1* (UP2101) and the known *MLH1* pLoF PP-SV (SMU080) showed reduced expression levels – with log_2_ fold change values of -1.36, -1.07 and -0.67, respectively (Supplementary Fig. 18A). Additionally, high expression of *COL4A2* in contrast to lack of expression of *COL4A1* in patient UP2039 (Supplementary Fig. 18B) concurs with our expected copy gain impact as a consequence of the former gene duplication (Supplementary Fig. 9). The near to average expression of ‘cautionary’ PP-SV impacting *PHC3* and *PRKACA* in patient SMU061, with normalised counts of 5863.56 *vs*. 6816.17 and 1689.19 *vs*. 1569.10, respectively (Supplementary Fig. 18C), provides further validation for our cautionary classification.

Furthermore, tumour-matched samples from PP-SV presenting patients were assessed for biallelic loss or a second somatic hit, both potential indicators of gene-relevant causality^49^. We used TITAN to infer for copy number (CN) loss as a prediction of loss of heterozygosity (LOH-CNL) due to deletion of wild-type allele, and CN neutral or gain LOH (LOH-CNN and LOH-CNG, respectively) due to accompanied duplication of the mutant allele^50^. After correction for tumour purity and ploidy (Supplementary Data 3), we identified LOH in five PP-SV presenting patients, including two with ClinVar validated pathogenic or likely pathogenic SVs and three presenting with ISUP 4-5 disease (Table 2). LOH-CNL was observed in a single patient SMU083 resulting in biallelic loss of *PIGN*. LOH-CNG was observed in two patients, indicating wild-type allele loss for *SLC3A1* and *SLC7A2* in patients SMU094 and KAL0054, respectively. LOH-CNN, indicating wild-type allele loss with amplification of tumour suppressor losses, was observed in *BCL2L11* and *BARD1* for patients KAL0101 and N0073.

Overall and irrespective of ancestry, patients with germline PP-SVs (14 African, 4 European) showed less oncogenic driver variants than non-PP-SV presenting cases (99 African, 53 European), although not statistically significant (250, range: 101-437 *vs* 316, range: 105–772). In addition, we found the same gene second hit by somatic CN alterations including CN loss impacting *SLC3A1*, *SLC7A2* and two cautionary fusion PP-SVs in in an African patient each (all ISUP GG $\ge \,$3 at diagnosis) and in turn CN gain impacting *OCA2*, *FOXP1* and *WASF1* in each of three African patients with advanced ISUP GG5 disease (Supplementary Table 9 and Table 2). Notably, the single European patient presenting with the cautionary PP-SV impacting *LTBP1*, with second hit somatic CN gain, underwent surgery at 54 years of age for ISUP GG5 disease.

## Discussion

ClinVar defined pathogenic (or likely pathogenic) SVs disrupting solute carrier family 3 member 1 (*SLC3A1*), OCA2 melanosomal transmembrane protein (*OCA2*) or phosphatidylinositol glycan anchor biosynthesis class N (*PIGN*) were observed in 3.5% (4/113) of African patients. Specifically, the *SLC3A1* intragenic exon DUP was identified in two patients presenting with ISUP GG4, while the *OCA2* and *PIGN* pLoF DELs presented in a single patient, each with ISUP GG5 and ISUP GG3 PCa, respectively (Table 2). Visually inspecting the three PP-SVs using Integrative Genomic Viewer^51^, *SLC3A1* DUP was found with three supporting read-pairs in sample N0001 (Supplementary Fig. 3), and split-reads and more than 40% increase in read depth comparing to $\pm$10 kb of the SV region in both samples (Supplementary Table 10), while *OCA2* and *PIGN* DELs were found with 16 and 6 supporting read-pairs respectively (Supplementary Fig. 1, 2), and have 44-51% reduction in read depth (Supplementary Table 10). *SLC3A1* is an amino acid transporter, which, through heterodimerisation with *SLC7A9* is responsible for cystine reabsorption through cationic and neutral amino acid exchange^52^. Mutations, including SVs, in *SCL3A1* are associated with cystinuria, an inherited disease that results in the formation of cystine stones in the kidney, with disease presentation suggested to require biallelic loss^53^. *SCL3A1* over-expression has been associated with enhanced tumourigenesis in breast cancer while blocking *SCL3A1* has suggestive therapeutic potential^39^. Taken together, it is notable that somatic LOH-CNG, with second hit CN loss, was observed for the younger of the two *SLC3A1* PP-SV presenting African patients (SMU094). *OCA2* is a pigmentation gene with inherited mutations associated with oculocutaneous albinism^54^. Polymorphisms have been associated with skin cancers^55^, as well as clinical response and survival in breast cancer patients having received neoadjuvant chemotherapy^56^. Notably, the ISUP GG5 presenting pLoF germline SV African patient also with a second hit somatic *OCA2* CN gain. Inherited *PIGN* mutations have been associated with multiple congenital anomalies-hypotonia-seizures syndrome and Fryns syndrome, with some mutations related to milder forms of clinical presentation^57,58^. *PIGN* is involved in the biosynthesis of glycosylphosphatidylinositol, which has been shown to suppress cancer chromosomal instability^37^ through PIGN complexed spindle assembly checkpoint regulation^38^, a common phenomenon in solid tumours^59^. Biallelic *PIGN* loss is tentatively predicted in the tumour of the older aged presenting African patient. While assumed pathogenic, an association between *SCL3A1, OCA2* or *PIGN* mutation and PCa is yet to be elucidated.

As our study is biased towards under-represented African patients, it is highly plausible that the majority of SVs detected are unlikely to be represented in ClinVar. As such, it is critical that we develop a best-fit workflow for PP-SV prediction. The four SV impact prediction tools used in this study were chosen based on the criteria of easy-to-use (either web-based or packed as software), providing pathogenicity scores or labels, accepting multiple SVs and covering all SV types. However, there are multiple factors to be taken into consideration when using SV impact prediction tools to establish potential pathogenicity, as different tools have limitations in applicable SV types, regions or diseases, as well as different scoring systems. While all tools can predict the impact of DELs and DUPs, StrVCTVRE is limited to DELs and DUPs in exonic regions. Besides predicting the simpler SVs, CADD-SV is capable of annotating INSs, POSTRE annotates INVs and TRAs, while PhenoSV is able to predict the impact of all these three types. POSTRE doesn’t work for all diseases or phenotypes. Therefore, combining multiple tools is necessary to cover all SV types and increase the confidence level. Another factor is the choice of threshold to establish pathogenicity. POSTRE and PhenoSV define the threshold of pathogenicity, but StrVCTVRE and CADD-SV are limited to scores and calling for thresholds to be established depending on individual study aims. In this study, we have decided the thresholds based on tools’ validated results from the database (90% sensitivity in ClinVar by StrVCTVRE^43^ and top 10% in gnomAD by CADD-SV^44^). When combining results from multiple tools, we found the requirement of passing thresholds of all four tools identified two PP-SV candidates (out of 1843 SVs) (Supplementary Table 4), with notable failure to identify the three ClinVar pathogenic/likely pathogenic SVs (Supplementary Table 6). As such, PP-SV candidate classification in this study required an SV to pass thresholds of at least two impact prediction tools, with disrupted genes requiring further clarification as hallmarks or drivers in cancer gene databases (MSigDB and COSMIC CGC).

Using our described workflow, 12 SVs were predicted as PP-SVs, identified in 7.0% (4/57) of European and 8.8% (10/113) of African patients, bringing the total of African patients presenting with a potential pathogenic SV to 12.4% (14/113). Remarkably, five of our African-specific PP-SVs included well-known pathogenic cancer genes and/or PCa tumour suppressor genes, including DNA damage response genes. Most notably, the DNA mismatch repair tumour suppressor gene mutL homologue 1 (*MLH1*) commonly mutated in Lynch Syndrome, including cases with PCa^60^, is a known candidate gene in PCa germline testing panels^20^. While PCa patients presenting with pathogenic *MLH1* mutations were reported to have significantly higher disease burden for African Americans^24^, here we found a dbVar known *MLH1* pLoF INV with around 11 supporting short read-pairs (Supplementary Fig. 12) in a 64-year-old African male presenting with ISUP GG4 at diagnosis and at the time of diagnosis no somatic LOH or a second gene hit. Not recognised as a PCa germline testing panel gene, forkhead box P1 (*FOXP1*) is an established PCa tumour suppressor driver gene, with CN loss increasing cell proliferation and migration, and poor prognosis^61^. Recently, we showed *FOXP1* to be equally impacted by predominantly CN loss in African compared with European-derived tumours (20% of 183 tumours)^18^. Here we found a germline inverted duplication impacting *FOXP1* with around 18 supporting read-pairs in two African patients (Supplementary Fig. 15). Notably, one African patient (UP2101) presented 10 years earlier than the cohort average receiving an ISUP GG5 diagnosis, however, this patient also presented with a second hit somatic *FOXP1* CN gain. Loss of the *BRAC1-*associated RING domain-1 (*BARD1*) DNA damage repair gene has been found to induce homologous recombination deficiency and increase the sensitivity to PARP inhibitor in PCa cell lines^62^. Here the unknown *BARD1* exon 5 DEL, supported by 10 read-pairs and with around 50% reduction in read depth comparing to $\pm$10 kb of the DEL region (Supplementary Fig. 8 and Supplementary Table 10), was identified in a 62-year-old African PCa patient with unknown pathology, with associated amplification of the deleted allele during tumourigenesis. While a paediatric cancer predisposing tumour suppressor gene commonly mutated in retinoblastoma and to a lesser extent osteosarcoma^63^, and less common as an adult cancer predisposing gene^64^, Retinoblastoma transcriptional corepressor 1 (*RB1*) is recognised as one of five most prevalent somatically mutated genes in metastatic cancers^65^, with *RB1* loss in prostate tumours associated with poor patient outcomes^66^. To the best of our knowledge, no germline potentially pathogenic *RB1* variant has been reported for PCa, which includes a pLoF INV of exon 24 with three supporting read-pairs (Supplementary Fig. 13) in a single ISUP GG3 diagnosed African patient. Lastly, we found a PP-SV in the tumour suppressor gene WASP family member 1 (*WASF1*) with loss previously associated with aggressive or metastatic lethal PCa^67^. A potentially pathogenic INV, previously reported at MAF of 9.6e-05 in Africans and resulting in *NR2E1-WASF1* fusion, was identified in a single African patient presenting at 70 years of age with ISUP GG5 PCa, showed 14 supporting read-pairs (Supplementary Fig. 14), with somatic hyper-amplification during tumourigenesis.

Other notable PP-SV DELs impacting tumour suppressor genes unknown to PCa include solute carrier family 7 member 2 (*SLC7A2*) and DnaJ heat shock protein family member C15 (*DNAJC15*). Knockdown of *SLC7A2* has been shown to promote viability, invasion and migration of ovarian cancer^68^ and enhance proliferation of non-small-cell lung cancer cells^69^, while *DNAJC15* has tumour suppressor behaviour in breast cancer^70^. Identified in two African patients presenting with ISUP GG5 disease, loss of *SLC7A2* exons 1 and 2, supported by 10 read-pairs and with around 50% reduction in read depth (Supplementary Fig. 5 and Supplementary Table 10) has previously been reported in African populations at MAF of 0.03 (Supplementary Data 1). Notably, the younger of the two patients showed loss of the wild-type allele during tumourigenesis. Specific to Europeans (MAF = 1.0e-04), loss of *DNAJC5* exon 4 supported by 13 read-pairs and with around 50% reduction in read depth (Supplementary Fig. 6 and Supplementary Table 10), was identified in a single European patient presenting for surgery at age 63 years with ISUP GG5 disease, yet appears to remain diploid during tumour development. While not associated with PCa, the loss of BCL2 like 11 (*BCL2L11*) and catenin alpha 1 (*CTNNA1*) has been identified as drivers of tumourigenesis and promoting invasion and metastasis of multiple cancers^71,72^. Unknown SVs include; *BCL2L11* pLoF DEL impacting exon 2 with more than 20 supporting read-pairs and with around 50% reduction in read depth (Supplementary Fig. 7 and Supplementary Table 10) was identified in a single African patient presenting at age 71 years with ISUP GG5 PCa, while pLoF TRA interrupting *CTNNA1* with more than 20 supporting read-pairs (Supplementary Fig. 16) in a single European patient presenting at age 59 years with ISUP GG5 PCa. Notably, the African *BCL1L11* PP-SV presenting patient showed further somatic LOH. Another currently unknown SV identified included the potentially pathogenic inter-chromosomal TRA with around 18 supporting read-pairs (Supplementary Fig. 17) leading to an adenylate kinase 8 (*AK8*)*-* dystonin (*DST*) fusion in a single European patient (ISUP GG1, 67 years). Although no associations have been made between PCa, higher expression of *DST* has been identified to promote pathogenesis and development of breast cancer, while *AK8* downregulation has been found to promote migration and invasion of uterine carcinosarcoma^73^.

Two identified PP-SVs have the potential to increase gene dosage of well-known oncogenes collagen type IV alpha 2 chain (*COL4A2*) and solute carrier family 2 member 5 (*SLC2A5*) through whole-gene and intra-genic exon duplication, respectively. Although not associated with PCa, *COL4A2* loss has been identified to inhibit triple-negative breast cancer cell proliferation and migration^74^ and its mutations as a risk factor for familial cerebrovascular disease^75^, while inactivation of *SLC2A5* has been found to inhibit cell proliferation and migration in multiple cancer cell lines^76^. The whole *COL4A2* DUP with more than 20 supporting read-pairs and more than 50% gain in read depth (Supplementary Fig. 9 and Supplementary Table 10) was identified in a single African patient (ISUP GG4, 71 years) and the exon 4 DUP in *SLC2A5* with more than 20 supporting read-pairs and more than 50% increase in read depth (Supplementary Fig. 11 and Supplementary Table 10) was identified in a single European patient (ISUP GG5, 70 years). No LOH or second hits were observed during tumorigenesis.

Using short-read sequencing data for SV calling and genotyping remains a potential limitation, appreciating that SVs in difficult-to-sequence regions may have been overlooked^77^. To ensure the highest possible accuracy of SV detection and population allele frequency estimation, we required high-confidence calls from two SV callers and high-quality genotype calls at both the population- and individual-level, while all PP-SVs were visually inspected. Due to a lack of available expression data, we were unable to validate the direct impact of identified PP-SVs and cautionary PP-SVs. While LOH or second hits in the developing tumours added further possible causality to several candidate genes, one cannot ignore that LOH can occur by chance. Conversely, we cannot exclude hypermethylation inactivation as a second hit for the remaining gene candidates. Additional study limitations related to our southern African cohort include (i) a lack of population-matched healthy controls or regionally relevant population-wide whole genome data, (ii) the on average older age at PCa presentation^23,78^ and lack of PCa knowledge as related to family history^79^, both criteria traditionally used for genetic testing, and (iii) a lack of African-relevant data in currently pathogenicity prediction databases. As a consequence of these illimitations, our study calls for further African-inclusive efforts and for the establishment of guidelines for pathogenic SV identification using both short and/or long-read sequencing approaches, making these methods accessible for routine multi-ancestral germline testing.

Here, we provide substantial evidence that inherited SVs may not only be contributing to PCa pathogenicity but also associated ancestry disparity. We observed three ClinVar-defined pathogenic or likely pathogenic PP-SVs (*SLC3A1*, *OCA2* and *PIGN*) and 12 predicted PP-SVs, including seven known SVs (*SLC7A2, DNAJC15*, *COL4A2*, *SLC2A5*, *WASF1*, *MLH1* and *RB1*), and five unknown SVs (*BCL2L11, BARD1, FOXP1*, *CTNNA1* and *AK8-DST*) of which patients presenting with *BCL2L11* and *FOXP1* SVs show associated loss of gene expression, suggesting that inherited SVs may constitute an under-appreciated contribution to PCa pathogenicity. Furthermore, the identification of African-private (8 known, 3 unknown) and European-private (2 known, 2 unknown) PP-SVs allows for further speculation with regard to associated racial disparities while improving the detection rate for PCa germline testing with SV inclusivity, and in turn raising limitations for African inclusion and associated clinical care.

## Methods

### Participant recruitment and ethics approval

Irrespective of country of origin, all individuals provided written and signed informed consent to participate in the study and publish. Conforming to the principles of the Helsinki Declaration, South African patients were recruited as part of the Southern African Prostate Cancer Study (SAPCS) with approval granted by the University of Pretoria Faculty of Health Sciences Research Ethics Committee (HREC, with US Federal-wide assurance FWA00002567 and IRB00002235 IORG0001762; #43/2010). In Australia, participant recruitment was approved by the St Vincent’s HREC (#SVH/12/231). As all patients underwent a prostate biopsy (South Africa) or surgery (Australia), all are assumed to be biologically male. Samples were shipped to the Garvan Institute of Medical Research and, subsequently the University of Sydney in accordance with institutional Material Transfer Agreements (MTAs), as well as additional Republic of South Africa Department of Health Export Permit (National Health Act 2003; J1/2/4/2 #1/12). This study was approved by the St. Vincent’s HREC (#SVH/15/227) for genomic interrogation. Additional IRB review and approval for genomic interrogation was granted by the Human Research Protection Office of the US Army Medical Research and Development Command E02371 (TARGET Africa) and E03280 (HEROIC PCaPH Africa1K).

### WGS data generation

To avoid technical and analytical biases, all samples (whole blood) were processed (beginning at DNA extraction), data generated and analysed within a single laboratory using a single computational pipeline^18,28^. In brief, whole-genome sequencing data were generated using Illumina HiSeq X Ten (21 cases) or NovoSeq (149 cases) instruments with 2 × 150 cycle paired-end mode at the Kinghorn Centre for Clinical Genomics (Garvan Institute of Medical Research, Australia). Following the BROAD’s best practice recommendations for “data pre-processing for variant discovery”, sequencing reads were aligned to GRCh38 reference genome with alternative contigs using scalable FASTQ-to-BAM (v2.0) workflow with default settings^80^. The mean depth of coverage for all samples were 45.9X (range 30.2–97.6X).

### Structural variant calling and high-confidence SV filtering

Germline SVs were called using Manta (v1.6.0)^81^ and GRIDSS (v2.13.3)^82,83^. SV types reported by Manta included DEL, tandem DUP, INS and adjacent breakends (BNDs) for a fusion junction with an inverted sequence or in an inter-chromosomal rearranged genome. Pairs of BND in inverted junctions were annotated as inversions (INV). Pairs of BND in different chromosomes were annotated as inter-chromosomal translocations (TRA). Conversely, GRIDSS reports BND for all fusion junctions resulting from any SV event. Simple SV types, defined as DEL, DUP, INS, INV and TRA, were assigned based on the strands and ALT field in VCF (modified from GRIDSS accompanied R script: simple-event-annotation.R).To obtain a high-confidence SV call set, we integrated call sets from Manta and GRIDSS and generated a concordant call set for each genome. Two SV calls were considered as concordant if they were reported as “PASS” by one of the two callers and have matching SV type and reported breakpoint positions within 200 bp of each other. *Bedtools pairtopair*^84^ was used to compare two call sets.

### Population-level genotyping and high-confidence genotype call filtering

We used Graphtyper2 (v2.7.5)^85^ to re-genotype SVs for all samples. Following published guidelines, we merged all high-confidence SV sets persample (individual VCFs) using svimmer (https://github.com/DecodeGenetics/svimmer) with default parameters. The individual VCFs were in the format of Manta VCFs, as Manta provides detailed information on the exact breakpoint sequence, which is the essential information required by Graphtyper2. We extracted all SVs with the “aggregate” model as suggested and obtained 57,096 SVs with “PASS” in the FILTER field in VCF. We also required SVs to have more than 50% of genotype calls as “PASS” (PASS_ratio $\ge$ 0.5 in INFO field), resulting in 42,966 SVs.

To further filter SV genotype calls on a per-sample basis, we set the SV genotype as missing if the genotype filter tag (FT) is not “PASS” for all SVs, except BND. For BND, as the FT tag is not available, we set the BND genotype with genotype quality (GQ) < 20 as missing. We then excluded SVs with genotype missingness rate > 20% in either African or European genomes, resulting in 33,340 SVs. We further removed 97 SVs with an allele frequency of 100%, indicating the difference of the sample genomes to reference genome. The allele frequency of each SV was then calculated based on the high-quality genotype calls only.

### Gene annotation and functional impact of SVs

All SVs were annotated against gene regions from the Ensembl human gene annotation file (GRCh38 assembly, release 108)^86^. As multiple transcripts can be available for a single gene, the Ensembl Canonical transcript was used (http://www.ensembl.org/info/genome/genebuild/canonical.html). By comparing the position of SV breakpoint with gene regions using bedtools^84^, we examined nine gene overlapping categories with gnomAD^32^, including potential Loss of Function (pLoF), Copy Gain (CG), Intragenic Exon DUP (IED), partial gene DUP, whole-gene INV, UTR SVs, promoter SVs, intronic SVs and intergenic SVs. In addition, we defined partial-exon DUP as both breakpoints contained within the same gene, while neither both within exons (pLoF) nor fully overlapped at least one exon (IED). Promoters were defined as a 1 kb window before each transcription start site on the transcribed strand. We labelled SVs as enhancer-disruptive if at least one breakpoint was contained within a gene’s enhancer, by comparing to GeneHancer^87^ regulatory elements regions. GeneHancer regulatory elements and gene interactions “double elite” subset was downloaded from UCSC Table geneHancerInteractionsDoubleElite [last updated 15/01/2019] from GeneHancer track for GRCh38. The transcript structure plots were generated based on Ensembl human gene annotation (GRCh38 assembly, release 108) using R package ggtranscript (v0.99.3)^88^. The sequencing depth of DEL or DUP regions and their $\pm$10 kb regions were calculated using samtools (v1.6) depth command^89^.

Short-read data detect the SV signatures from aligned reads around the SV breakpoints, and is hard to capture the whole large SVs^90^. Therefore, we restricted the disrupted genes of SVs greater than 1Mbp to be genes overlapped by SV breakpoints for downstream analysis.

### Identification of dbVar concordance and unknnown SVs

The NCBI’s database of human genomic structural variation (dbVar) [last updated 30/10/2023]^91^ were used to identify dbVar concordance and unknown SVs. The dbVar database included a total of 6,476,337 unique SVs, including 86,686 SVs with interpretations of their significance to disease in the ClinVar database^92^. Structural variants concordant to dbVar SVs were defined as having both breakpoints within 200 bases of dbVar-defined SV breakpoints. The ancestry-related variant allele frequency of SVs (Supplementary Data 1) were derived from dbVar pages of SVs or VCFs uploaded by different dbVar studies to dbVar’s FTP site.

### Pathogenicity prediction

The pathogenicity of SVs were predicted through prediction tools StrVCTVRE^43^, CADD-SV^44^, POSTRE^45^ and PhenoSV^46^. StrVCTVRE only scores the deleteriousness of DEL and DUP overlapping one or more exons, CADD-SV scores DEL, DUP and INS, POSTRE predicts the impact of DEL, DUP, INV and TRA, and PhenoSV works for all five SV types. As POSTRE only accepts genome coordinates on reference genome Hg19, the *liftOver* function from *rtracklayer* package in R was used to lift SV coordinates from Hg38 to Hg19. As suggested by StrVCTVRE, the ClinVar 90% sensitivity threshold (0.37) was used to define potentially pathogenic SVs. The scaled CADD-SV scores range from 0 (potentially benign) to 48 (potentially pathogenic), indicating the position of the input SV within the gnomAD-SV score distribution. The threshold of 10 for the CADD-SV score was used to establish potential pathogenicity, corresponding to the top 10% score observed in gnomAD-SV. The threshold of 0.8 and 0.5 for POSTRE and PhenoSV score, respectively, was used in this study, which is the threshold of pathogenicity labelling defined by POSTRE and PhenoSV.

The hallmark gene sets and oncogenic signature gene sets were downloaded from the Human Molecular Signature Database (MSigDB v2023.1)^47^. The MSigDB oncogenic signature gene sets included genes representing signatures of cellular pathways which are often dis-regulated in cancer. Cancer-driver genes were downloaded from the COSMIC Cancer Gene Census (GRCh38 COSMIC v98, downloaded 26/09/2023).

### PP-SV allele frequency predictions

The VAF of each PP-SV was calculated as the fraction of altered sequencing reads within each SV region. Three types of altered sequencing reads were considered, including the reduced or increased sequencing reads due to DEL or DUP, discordantly aligned read-pairs and split reads. For DEL and DUP, the altered read count was calculated as the average read depth difference between the SV region and its $\pm$10 kb regions. The total read count of DEL was measured as the average read depth of $\pm$10 kbp region to SV region, and that for DUP was the average read depth of SV region. For INV and TRA, the altered reads, including discordantly aligned reads and split-reads, within $\pm$150 bp of each breakpoint were manually inspected using IGV. The total read count was measured as average sequencing read depth in $\pm$150 bp region to INV or TRA breakpoints, calculated using samtools (v1.6) *depth* command. The SV VAF can be underestimated due to one read can show both discordant read-pair and split-reads, while only counted once. In addition, non-reference read-pairs is possible to be aligned properly in the SV region. In addition, whole genome data was made available for 49 Southern Bantu-matched disease-free individuals for further PP-SV candidate gene interrogation for comparative analyses. SV calling and genotyping, and high-confidence SV filtering were implemented exactly the same as PCa patients.

### Patient matched RNA-seq analysis for gene expression

Whole blood was available for 20 African patients (KAL047, KAL054, KAL061, KAL0101, N0053, N0056, SMU061, SMU080, SMU094, SMU109, SMU141, UP2035, UP2039, UP2092, UP2093, UP2100, UP2101, UP2119, UP2159, and UP2187) from which total RNA was extracted using the QIAamp RNA Blood Mini Kit (Qiagen, Hilden, Germany) and sequenced to generate an average of 108 million paired-end reads per sample. Quality control was performed with raw RNA-seq FASTQ files using FastQC^93^, with summary reports generated by aggregating individual reports with MultiQC^94^. To remove ribosomal RNA (rRNA) contamination, we filtered the reads using SortMeRNA^95^ with SILVA and RFAM databases^96,97^. A human reference genome index was generated using GRCh38 primary assembly GENCODE v47 (https://www.gencodegenes.org/human/). Reads were aligned, and gene-level counts were quantified using the STAR aligner^98^, resulting in an average genome coverage of 9x. Gene count matrices were imported into RStudio for downstream analysis. Genes with low expression were filtered out using DESeq2, retaining only those with more than 10 counts in at least three samples^99^. To mitigate potential globin mRNA contamination reads aligning to globin genes were excluded from further downstream analyses^100,101^. Count normalisation was performed using the median of ratios method in DESeq2^99^. Gene-specific expressions were plotted for PP-SV patients against the non-PP-SV controls.

### Biallelic loss and somatic second hit identification in PP-SV presenting patients

LOH was inferred by TitanCNA snake workflow (TITAN) v1.17.1^18,50^. In brief, tumour purity and ploidy corrected copy number status was inferred from matched tumour WGS data of PP-SV presenting patients by TITAN. The gene region with the adjusted discrete copy number of one allele as zero was considered to have LOH status. Depending on the copy number of another allele (1,2 or $\ge$2), TITAN predicted LOH status to hemizygous LOH, copy neutral LOH and amplification LOH, respectively (Supplementary Data 3).

The number of somatic oncogenic driver variants in all patients were obtained from our previous study^18^, accounted for coding or noncoding driver mutation, significantly recurrent breakpoint and gene-level copy number amplification or deletion. In addition, all oncogenic driver variants were assessed for their presence in PP-SV presenting patients, as second somatic hit to PP-SV disrupted genes.

### Inclusion and Ethics Statement

Local researchers are included in this research as co-directors for the Southern African Prostate Cancer Study (SAPCS) from study design to interpretation, including data ownership via leadership on the SAPCS Data Access Committee (DAC), which also includes meeting all criteria for full authorship. The SAPCS Directorship includes clinical (M.S.R.B., University of Pretoria, South Africa), urological (S.B.A.M., Sefako Magatho Health Sciences University, South Africa) and scientific leaders (V.M.H., which includes affiliation at University of Pretoria, South Africa). Southern African data and material for RNA-sequencing data generation was accessed via the SAPCS DAC and through a fully executed collaborative research agreement (CRA), which includes shared funding.

### Reporting summary

Further information on research design is available in the Nature Portfolio Reporting Summary linked to this article.

## Supplementary information

Supplementary Information

Peer Review File

Description of Additional Supplementary Files

Supplementary Data 1

Supplementary Data 2

Supplementary Data 3

Reporting Summary

## Source data

Source Data

## Data Availability

Access to published whole genome sequence data (Jaratlerdsiri et al. ^18^) was made available via Data Access Committee (DAC) approval as outlined under the European Genome-Phenome Archive (EGA) [https://ega-archive.org] project-specific access policies under overarching study EGAS00001006425, which includes the Southern African Prostate Cancer Study (SAPCS) Dataset at EGAD00001009067 and Garvan/St Vincent’s Prostate Cancer Database at EGAD00001009066. The mapped RNA-sequencing data in BAM format have been deposited under study EGAS50000000702 with accession number EGAD50000000982. Access to the RNA-sequencing data may be requested via the SAPCS DAC and will be made available to researchers with appropriate feasibility and corresponding ethics approvals to ensure the safeguarding of patient genomic information (contact V.M.H.). Restrictions include (i) No transfer to third parties allowed, (ii) acknowledgement of the SAPCS in publications/presentations, (iii) a report of the results of the research to be provided to DAC after completion (or when requested), (iv) researchers cannot utilise the data for commercial purposes, or any other purposes not approved by the DAC, and (v) approval will not be given that excludes other researchers from accessing data. Data currently being used for capacity building in under-resourced studies across Sub-Saharan Africa will be given priority and at times, may be granted time-limited exclusive rights for no more than a two-year period. SV and related annotation data supporting the findings of this study are available within the main text, Supplementary information and source data. Previously published SV sites and their population variant allele frequencies are available in the dbVar database [https://www.ncbi.nlm.nih.gov/dbvar]^91^, gene regions are available in the ENSEMBL database [https://www.ensembl.org]^86^, gene sets at MSigDB^47^ [https://www.gsea-msigdb.org/gsea/index.jsp] and cancer driver gene sets at COSMIC CGC^48^ [https://cancer.sanger.ac.uk/census]. Source data are provided in this paper.

## Code availability

Software and scripts for DNA sequence read data collection are available at GitHub (https://github.com/Sydney-Informatics-Hub/Bioinformatics). FastQC 0.11.7, MultiQC 1.25.1, Trimmomatic 0.38, SortMeRNA 4.3.3 and STAR aligner 2.7.1a for RNA sequence data collection. The scripts for sequence read alignment and quality control are available on GitHub (https://github.com/Sydney-Informatics-Hub/Bioinformatics). All computational code for SV call set comparison and integration are available at GitHub (https://github.com/tgong1/StructuralVariantUtil)^102^.

## References

[1] Bray, F. et al. Global cancer statistics 2022: GLOBOCAN estimates of incidence and mortality worldwide for 36 cancers in 185 countries. *Cancer J. Clin**.* **74**, 229–263 (2024). 10.3322/caac.21834 38572751

[2] Hjelmborg, J. B, et al. The heritability of prostate cancer in the nordic twin study of cancer. *Cancer Epidemiol. Biomarkers Prev.* **23**, 2303–2310 (2014). 24812039 10.1158/1055-9965.EPI-13-0568 PMC4221420

[3] Smith, Z. L., Eggener, S. E. & Murphy, A. B. African-American prostate cancer disparities. *Curr. Urol. Rep.* **18**, 81 (2017). 28808871 10.1007/s11934-017-0724-5

[4] Taitt, H. E. Global trends and prostate cancer: A review of incidence, detection, and mortality as influenced by race, rthnicity, and geographic location. *Am. J. Mens Health* **12**, 1807–1823 (2018). 30203706 10.1177/1557988318798279 PMC6199451

[5] Giaquinto, A. N. et al. Cancer statistics for African American/Black People 2022. *Cancer J. Clin.* **72**, 202–229 (2022). 10.3322/caac.21718 35143040

[6] Mahal, B. A. et al. Prostate cancer racial disparities: A systematic review by the prostate cancer foundation panel. *Eur. Urol. Oncol.* **5**, 18–29 (2022). 34446369 10.1016/j.euo.2021.07.006

[7] Darst, B. F. et al. Combined effect of a polygenic risk score and rare genetic variants on prostate cancer risk. *Eur. Urol.* **80**, 134–138 (2021). 33941403 10.1016/j.eururo.2021.04.013 PMC8286329

[8] Seibert, T. M. et al. Genetic risk prediction for prostate cancer: Implications for early detection and prevention. *Eur. Urol.* **83**, 241–248 (2023). 36609003 10.1016/j.eururo.2022.12.021

[9] de Bono, J. et al. Olaparib for metastatic castration-resistant prostate cancer. *N. Engl. J. Med.* **382**, 2091–2102 (2020). 32343890 10.1056/NEJMoa1911440

[10] Abida, W. et al. Rucaparib in men with metastatic castration-resistant prostate cancer harboring a BRCA1 or BRCA2 gene alteration. *J. Clin. Oncol.* **38**, 3763–3772 (2020). 32795228 10.1200/JCO.20.01035 PMC7655021

[11] Lozano, R. et al. Genetic aberrations in DNA repair pathways: a cornerstone of precision oncology in prostate cancer. *Br. J. Cancer* **124**, 552–563 (2021). 33106584 10.1038/s41416-020-01114-x PMC7851123

[12] National Comprehensive Cancer Network Clinical Guidelines in Oncology (NCCN Guidelines®): Prostate Cancer (Version 4.2023). 10.6004/jnccn.2023.0050 37856213

[13] Giri, V. N. et al. Implementation of germline testing for prostate cancer: Philadelphia prostate cancer consensus conference 2019. *J. Clin. Oncol.* **38**, 2798–2811 (2020). 32516092 10.1200/JCO.20.00046 PMC7430215

[14] Briggs, L. G. et al. Racial differences in germline genetic testing for prostate cancer: A systematic review. *J. Oncol. Pract.* **19**, e784–e793 (2023). 10.1200/OP.22.00634 36649495

[15] Mahal, B. A. et al. Racial differences in genomic profiling of prostate cancer. *N. Engl. J. Med.* **383**, 1083–1085 (2020). 32905685 10.1056/NEJMc2000069 PMC8971922

[16] Valle, L. F. et al. Actionable genomic alterations in prostate cancer among black and white united states veterans. *Oncologist* **28**, e473–e477 (2023). 37084789 10.1093/oncolo/oyad042 PMC10243786

[17] White, J. A. et al. Whole-exome sequencing of Nigerian prostate tumors from the prostate cancer transatlantic consortium (CaPTC) reveals DNA repair genes associated with African ancestry. *Cancer Res. Commun.* **2**, 1005–1016 (2022). 36922933 10.1158/2767-9764.CRC-22-0136 PMC10010347

[18] Jaratlerdsiri, W. et al. African-specific molecular taxonomy of prostate cancer. *Nature* **609**, 552–559 (2022). 36045292 10.1038/s41586-022-05154-6 PMC9477733

[19] Giri, V. N., Hartman, R., Pritzlaff, M., Horton, C. & Keith, S. W. Germline variant spectrum among African American men undergoing prostate cancer germline testing: Need for equity in genetic testing. *J. Precis. Oncol.* **6**, e2200234 (2022). 10.1200/PO.22.00234 PMC9200399 35666082

[20] Gheybi, K. et al. Evaluating germline testing panels in Southern African males with advanced prostate cancer. *J. Natl. Compr. Cancer Netw.* **21**, 289–296.e283 (2023). 10.6004/jnccn.2022.7097 36898365

[21] Soh, P. X. Y. & Hayes, V. M. Common genetic variants associated with prostate cancer risk: The need for African inclusion. *Eur. Urol.* **84**, 22–24 (2023). 37100647 10.1016/j.eururo.2023.04.006

[22] Soh, P. X. Y. et al. Prostate cancer genetic risk and associated aggressive disease in men of African ancestry. *Nat. Commun.* **14**, 8037 (2023). 38052806 10.1038/s41467-023-43726-w PMC10697980

[23] Tindall, E. A. et al. Clinical presentation of prostate cancer in Black South Africans. *Prostate* **74**, 880–891 (2014). 24723425 10.1002/pros.22806 PMC4135056

[24] Matejcic, M. et al. Pathogenic variants in cancer predisposition genes and prostate cancer risk in men of African ancestry. *J. Precis. Oncol.* **4**, 32–43 (2020). 10.1200/PO.19.00179 PMC7442213 32832836

[25] Pritchard, C. C. et al. Inherited DNA-repair gene mutations in men with metastatic prostate cancer. *N. Engl. J. Med.* **375**, 443–453 (2016). 27433846 10.1056/NEJMoa1603144 PMC4986616

[26] The Cancer Genome Atlas Research N. The molecular taxonomy of primary prostate cancer. *Cell* **163**, 1011–1025 (2015). 26544944 10.1016/j.cell.2015.10.025 PMC4695400

[27] Ryan, M. J. & Bose, R. Genomic alteration burden in advanced prostate cancer and therapeutic implications. *Front. Oncol.* **9**, 1287–1287 (2019). 31824860 10.3389/fonc.2019.01287 PMC6882947

[28] Gong, T. et al. Genome-wide interrogation of structural variation reveals novel African-specific prostate cancer oncogenic drivers. *Genome Med.* **14**, 100 (2022). 36045381 10.1186/s13073-022-01096-w PMC9434886

[29] Pagnamenta, A. T. et al. Structural and non-coding variants increase the diagnostic yield of clinical whole genome sequencing for rare diseases. *Genome Med.* **15**, 94 (2023). 37946251 10.1186/s13073-023-01240-0 PMC10636885

[30] Thibodeau, M. L. et al. Improved structural variant interpretation for hereditary cancer susceptibility using long-read sequencing. *Genet Med.* **22**, 1892–1897 (2020). 32624572 10.1038/s41436-020-0880-8 PMC7605438

[31] Dixon, K. et al. Defining the heterogeneity of unbalanced structural variation underlying breast cancer susceptibility by nanopore genome sequencing. *Eur. J. Hum. Genet.* **31**, 602–606 (2023). 36797466 10.1038/s41431-023-01284-1 PMC10172360

[32] Collins, R. L. et al. A structural variation reference for medical and population genetics. *Nature* **581**, 444–451 (2020). 32461652 10.1038/s41586-020-2287-8 PMC7334194

[33] Park, S.-J., Yoon, B.-H., Kim, S.-K. & Kim, S.-Y. GENT2: an updated gene expression database for normal and tumor tissues. *BMC Med. Genomics* **12**, 101 (2019). 31296229 10.1186/s12920-019-0514-7 PMC6624177

[34] Pellikaan, K. et al. Malignancies in prader-willi syndrome: results from a large international cohort and literature review. * J. Clin. Endocrinol. Metab.* **108**, e1720–e1730 (2023). 37267430 10.1210/clinem/dgad312 PMC10655548

[35] De Summa, S. et al. The genetic germline background of single and multiple primary melanomas. *Front. Mol. Biosci.* **7**, 555630 (2020). 33748184 10.3389/fmolb.2020.555630 PMC7973206

[36] Cole-Clark, D. et al. An initial melanoma diagnosis may increase the subsequent risk of prostate cancer: Results from the New South Wales Cancer Registry. *Sci. Rep.* **8**, 7167 (2018). 29740153 10.1038/s41598-018-25408-6 PMC5940665

[37] Burrell, R. A. et al. Replication stress links structural and numerical cancer chromosomal instability. *Nature* **494**, 492–496 (2013). 23446422 10.1038/nature11935 PMC4636055

[38] Teye, E. K. et al. PIGN spatiotemporally regulates the spindle assembly checkpoint proteins in leukemia transformation and progression. *Sci. Rep.* **11**, 19022 (2021). 34561473 10.1038/s41598-021-98218-y PMC8463542

[39] Jiang, Y. et al. Cysteine transporter SLC3A1 promotes breast cancer tumorigenesis. *Theranostics* **7**, 1036–1046 (2017). 28382174 10.7150/thno.18005 PMC5381264

[40] Auton, A. et al. A global reference for human genetic variation. *Nature* **526**, 68–74 (2015). 26432245 10.1038/nature15393 PMC4750478

[41] Byrska-Bishop, M. et al. High-coverage whole-genome sequencing of the expanded 1000 Genomes Project cohort including 602 trios. *Cell* **185**, 3426–3440.e3419 (2022). 36055201 10.1016/j.cell.2022.08.004 PMC9439720

[42] Jun G., et al. Structural variation across 138,134 samples in the TOPMed consortium. Preprint at 10.1101/2023.01.25.525428 (2023).

[43] Sharo, A. G., Hu, Z., Sunyaev, S. R. & Brenner, S. E. StrVCTVRE: A supervised learning method to predict the pathogenicity of human genome structural variants. * Am. J. Hum. Genet.* **109**, 195–209 (2022). 35032432 10.1016/j.ajhg.2021.12.007 PMC8874149

[44] Kleinert, P. & Kircher, M. A framework to score the effects of structural variants in health and disease. *Genome Res.* **32**, 766–777 (2022). 35197310 10.1101/gr.275995.121 PMC8997355

[45] Sánchez-Gaya, V. & Rada-Iglesias, A. POSTRE: a tool to predict the pathological effects of human structural variants. *Nucleic Acids Res.* **51**, e54–e54 (2023). 36999617 10.1093/nar/gkad225 PMC10201441

[46] Xu, Z., Li, Q., Marchionni, L. & Wang, K. PhenoSV: interpretable phenotype-aware model for the prioritization of genes affected by structural variants. *Nat. Commun.* **14**, 7805 (2023). 38016949 10.1038/s41467-023-43651-y PMC10684511

[47] Liberzon, A. et al. The molecular signatures database (MSigDB) hallmark gene set collection. *Cell Syst.* **1**, 417–425 (2015). 26771021 10.1016/j.cels.2015.12.004 PMC4707969

[48] Sondka, Z. et al. The COSMIC Cancer Gene Census: describing genetic dysfunction across all human cancers. *Nat. Rev. Cancer* **18**, 696–705 (2018). 30293088 10.1038/s41568-018-0060-1 PMC6450507

[49] Srinivasan, P. et al. The context-specific role of germline pathogenicity in tumorigenesis. *Nat. Genet.* **53**, 1577–1585 (2021). 34741162 10.1038/s41588-021-00949-1 PMC8957388

[50] Ha, G. et al. TITAN: inference of copy number architectures in clonal cell populations from tumor whole-genome sequence data. *Genome Res.* **24**, 1881–1893 (2014). 25060187 10.1101/gr.180281.114 PMC4216928

[51] Robinson, J. T., Thorvaldsdottir, H., Wenger, A. M., Zehir, A. & Mesirov, J. P. Variant Review with the Integrative Genomics Viewer. *Cancer Res.* **77**, e31–e34 (2017). 29092934 10.1158/0008-5472.CAN-17-0337 PMC5678989

[52] Nagamori, S. et al. Novel cystine transporter in renal proximal tubule identified as a missing partner of cystinuria-related plasma membrane protein rBAT/SLC3A1. *Proc. Natl. Acad. Sci. USA* **113**, 775–780 (2016). 26739563 10.1073/pnas.1519959113 PMC4725474

[53] Martell, H. J. et al. Associating mutations causing cystinuria with disease severity with the aim of providing precision medicine. *BMC Genomics* **18**, 550 (2017). 28812535 10.1186/s12864-017-3913-1 PMC5558187

[54] Okulicz, J. F., Shah, R. S., Schwartz, R. A. & Janniger, C. K. Oculocutaneous albinism. *J. Eur. Acad. Dermatol. Venereol.* **17**, 251–256 (2003). 12702061 10.1046/j.1468-3083.2003.00767.x

[55] Roberts, M. R., Asgari, M. M. & Toland, A. E. Genome-wide association studies and polygenic risk scores for skin cancer: clinically useful yet? *Br. J. Dermatol.* **181**, 1146–1155 (2019). 30908599 10.1111/bjd.17917 PMC6761051

[56] Li, X. P. et al. OCA2 rs4778137 polymorphism predicts survival of breast cancer patients receiving neoadjuvant chemotherapy. *Gene* **651**, 161–165 (2018). 29409738 10.1016/j.gene.2018.01.100

[57] Fleming, L. et al. Genotype-phenotype correlation of congenital anomalies in multiple congenital anomalies hypotonia seizures syndrome (MCAHS1)/PIGN-related epilepsy. *Am. J. Med. Genet. A***170a**, 77–86 (2016). 26394714 10.1002/ajmg.a.37369 PMC4886552

[58] Jezela-Stanek, A., Mierzewska, H. & Szczepanik, E. Vertical nystagmus as a feature of PIGN-related glycosylphosphatidylinositol biosynthesis defects. *Clin. Neurol. Neurosurg.* **196**, 106033 (2020). 32585529 10.1016/j.clineuro.2020.106033

[59] Lengauer, C., Kinzler, K. W. & Vogelstein, B. Genetic instabilities in human cancers. *Nature* **396**, 643–649 (1998). 9872311 10.1038/25292

[60] Haraldsdottir, S. et al. Prostate cancer incidence in males with Lynch syndrome. *Genet. Med.* **16**, 553–557 (2014). 24434690 10.1038/gim.2013.193 PMC4289599

[61] Cai, H. et al. In Vivo Application of CRISPR/Cas9 revealed implication of Foxa1 and Foxp1 in prostate cancer proliferation and epithelial plasticity. *Cancers* **14**, 4381 (2022). 36139541 10.3390/cancers14184381 PMC9496785

[62] Dillon, K. M. et al. PALB2 or BARD1 loss confers homologous recombination deficiency and PARP inhibitor sensitivity in prostate cancer. *Npj Precis. Oncol.* **6**, 49 (2022). 35768576 10.1038/s41698-022-00291-7 PMC9242979

[63] Zhang, J. et al. Germline mutations in predisposition genes in pediatric cancer. *N. Engl. J. Med.* **373**, 2336–2346 (2015). 26580448 10.1056/NEJMoa1508054 PMC4734119

[64] Huang, K.-l et al. Pathogenic germline variants in 10,389 adult cancers. *Cell* **173**, 355–370 (2018). 29625052 10.1016/j.cell.2018.03.039 PMC5949147

[65] Robinson, D. R. et al. Integrative clinical genomics of metastatic cancer. *Nature* **548**, 297–303 (2017). 28783718 10.1038/nature23306 PMC5995337

[66] Abida, W. et al. Genomic correlates of clinical outcome in advanced prostate cancer. *Proc. Natl. Acad. Sci. USA* **116**, 11428–11436 (2019). 31061129 10.1073/pnas.1902651116 PMC6561293

[67] Sowalsky, A. G. et al. Loss of Wave1 gene defines a subtype of lethal prostate cancer. *Oncotarget* **6**, 12383–12391 (2015). 25906751 10.18632/oncotarget.3564 PMC4494945

[68] Sun, T., Bi, F., Liu, Z. & Yang, Q. SLC7A2 serves as a potential biomarker and therapeutic target for ovarian cancer. *Aging* **12**, 13281–13296 (2020). 32647070 10.18632/aging.103433 PMC7377849

[69] Jiang, S. et al. Lower SLC7A2 expression is associated with enhanced multidrug resistance, less immune infiltrates and worse prognosis of NSCLC. *Cell Commun. Signal.* **21**, 9 (2023). 36639771 10.1186/s12964-022-01023-x PMC9838041

[70] Alessandrini, F., Pezzè, L., Menendez, D., Resnick, M. A. & Ciribilli, Y. ETV7-Mediated DNAJC15 repression leads to doxorubicin resistance in breast cancer cells. *Neoplasia* **20**, 857–870 (2018). 30025229 10.1016/j.neo.2018.06.008 PMC6077117

[71] Huang, J. et al. The role of CTNNA1 in malignancies: An updated review. *J. Cancer* **14**, 219–230 (2023). 36741258 10.7150/jca.79236 PMC9891874

[72] Zhang, H. et al. Onco-miR-24 regulates cell growth and apoptosis by targeting BCL2L11 in gastric cancer. *Protein Cell* **7**, 141–151 (2016). 26758252 10.1007/s13238-015-0234-5 PMC4742383

[73] Huang, R. et al. Co-expression analysis of genes and tumor-infiltrating immune cells in metastatic uterine carcinosarcoma. *Reprod. Sci.* **28**, 2685–2698 (2021). 33905082 10.1007/s43032-021-00584-5

[74] JingSong, H. et al. siRNA-mediated suppression of collagen type iv alpha 2 (COL4A2) mRNA inhibits triple-negative breast cancer cell proliferation and migration. *Oncotarget* **8**, 2585–2593 (2017). 27906681 10.18632/oncotarget.13716 PMC5356825

[75] Verbeek, E. et al. COL4A2 mutation associated with familial porencephaly and small-vessel disease. *Eur. J. Hum. Genet.* **20**, 844–851 (2012). 22333902 10.1038/ejhg.2012.20 PMC3400734

[76] Groenendyk, J. et al. Loss of the fructose transporter SLC2A5 inhibits cancer cell migration. *Front. Cell Dev. Biol.* **10**, 896297 (2022). 36268513 10.3389/fcell.2022.896297 PMC9578049

[77] Porubsky, D. & Eichler, E. E. A 25-year odyssey of genomic technology advances and structural variant discovery. *Cell* **187**, 1024–1037 (2024). 10.1016/j.cell.2024.01.002 PMC10932897 38290514

[78] Gheybi, K. et al. Linking African ancestral substructure to prostate cancer health disparities. *Sci. Rep.* **13**, 20909 (2023). 38017150 10.1038/s41598-023-47993-x PMC10684577

[79] Hayes, V. M. et al. Health equity research outcomes and improvement consortium prostate cancer health precision Africa1K: Closing the health equity gap through rural community inclusion. *J. Urol. Oncol.* **22**, 144–149 (2024).

[80] Sadsad, R., Samaha, G. & Chew, T. Fastq-to-bam @ NCI-Gadi [Internet].). WorkflowHub (2021).

[81] Chen, X. et al. Manta: rapid detection of structural variants and indels for germline and cancer sequencing applications. *Bioinformatics* **32**, 1220–1222 (2016). 26647377 10.1093/bioinformatics/btv710

[82] Cameron, D. L. et al. GRIDSS: sensitive and specific genomic rearrangement detection using positional de Bruijn graph assembly. *Genome Res.* **27**, 2050–2060 (2017). 29097403 10.1101/gr.222109.117 PMC5741059

[83] Cameron, D. L. et al. GRIDSS2: comprehensive characterisation of somatic structural variation using single breakend variants and structural variant phasing. *Genome Biol.* **22**, 202 (2021). 34253237 10.1186/s13059-021-02423-x PMC8274009

[84] Quinlan, A. R. & Hall, I. M. BEDTools: a flexible suite of utilities for comparing genomic features. *Bioinformatics* **26**, 841–842 (2010). 20110278 10.1093/bioinformatics/btq033 PMC2832824

[85] Eggertsson, H. P. et al. GraphTyper2 enables population-scale genotyping of structural variation using pangenome graphs. *Nat. Commun.* **10**, 5402 (2019). 31776332 10.1038/s41467-019-13341-9 PMC6881350

[86] Cunningham, F. et al. Ensembl 2022. *Nucleic Acids Res.* **50**, D988–D995 (2022). 34791404 10.1093/nar/gkab1049 PMC8728283

[87] Fishilevich, S. et al. GeneHancer: genome-wide integration of enhancers and target genes in GeneCards. *Database* **2017**, bax028 (2017). 28605766 10.1093/database/bax028 PMC5467550

[88] Gustavsson, E. K., Zhang, D., Reynolds, R. H., Garcia-Ruiz, S. & Ryten, M. ggtranscript: an R package for the visualization and interpretation of transcript isoforms using ggplot2. *Bioinformatics* **38**, 3844–3846 (2022). 35751589 10.1093/bioinformatics/btac409 PMC9344834

[89] Danecek, P. et al. Twelve years of SAMtools and BCFtools. *Gigascience* **10**, giab008 (2021). 33590861 10.1093/gigascience/giab008 PMC7931819

[90] Gong, T., Hayes, V. M. & Chan, E. K. F. Detection of somatic structural variants from short-read next-generation sequencing data. *Brief. Bioinform.* **22**, bbaa056 (2021). 32379294 10.1093/bib/bbaa056 PMC8138798

[91] Lappalainen, I. et al. DbVar and DGVa: public archives for genomic structural variation. *Nucleic Acids Res.* **41**, D936–D941 (2013). 23193291 10.1093/nar/gks1213 PMC3531204

[92] Landrum, M. J. et al. ClinVar: improving access to variant interpretations and supporting evidence. *Nucleic Acids Res.* **46**, D1062–D1067 (2018). 29165669 10.1093/nar/gkx1153 PMC5753237

[93] Andrews, S. FastQC: a quality control tool for high throughput sequence data. Available at: http://www.bioinformatics.babraham.ac.uk/projects/fastqc (2010).

[94] Ewels, P., Magnusson, M., Lundin, S. & Käller, M. MultiQC: summarize analysis results for multiple tools and samples in a single report. *Bioinformatics* **32**, 3047–3048 (2016). 27312411 10.1093/bioinformatics/btw354 PMC5039924

[95] Kopylova, E., Noé, L. & Touzet, H. SortMeRNA: fast and accurate filtering of ribosomal RNAs in metatranscriptomic data. *Bioinformatics* **28**, 3211–3217 (2012). 23071270 10.1093/bioinformatics/bts611

[96] Quast, C. et al. The SILVA ribosomal RNA gene database project: improved data processing and web-based tools. *Nucleic Acids Res.* **41**, D590–D596 (2013). 23193283 10.1093/nar/gks1219 PMC3531112

[97] Kalvari, I. et al. Rfam 14: expanded coverage of metagenomic, viral and microRNA families. *Nucleic Acids Res.* **49**, D192–D200 (2021). 33211869 10.1093/nar/gkaa1047 PMC7779021

[98] Dobin, A. et al. STAR: ultrafast universal RNA-seq aligner. *Bioinformatics* **29**, 15–21 (2013). 23104886 10.1093/bioinformatics/bts635 PMC3530905

[99] Love, M. I., Huber, W. & Anders, S. Moderated estimation of fold change and dispersion for RNA-seq data with DESeq2. *Genome Biol.* **15**, 550 (2014). 25516281 10.1186/s13059-014-0550-8 PMC4302049

[100] Sheerin, D. et al. Identification and control for the effects of bioinformatic globin depletion on human RNA-seq differential expression analysis. *Scientific Rep.* **13**, 1859 (2023). 10.1038/s41598-023-28218-7 PMC9892020 36725870

[101] Orcel, E. et al. A single workflow for multi-species blood transcriptomics. *BMC Genomics* **25**, 282 (2024). 38493105 10.1186/s12864-024-10208-2 PMC10944614

[102] Gong T. StructuralVariantUtil. *GitHub*, 10.5281/zenodo.14642422 (2024).

