# Supplementary material for: Rare pathogenic structural variants show potential to enhance prostate cancer germline testing for African men
Source: Nat Commun. 2025 Mar 10;16:2400. doi: 10.1038/s41467-025-57312-9 (PMC11893795; doi:10.1038/s41467-025-57312-9)
Supplement: Supplementary file 1 — Supplementary Information [file 41467_2025_57312_MOESM1_ESM.pdf]

Rare pathogenic structural variants show potential to enhance prostate cancer germline testing for African men

Tingting Gong, Jue Jiang, Korawich Uthayopas, M.S. Riana Bornman, Kazzem Gheybi, Phillip D. Stricker, Joachim Weischenfeldt, Shingai B. A. Mutambirwa, Weerachai Jaratlerdsiri and Vanessa M. Hayes

Supplementary Information

Supplementary Tables

Supplementary Table 1. Clinical and pathological characteristics of 170 prostate cancer patients included in this study

Variables		Prostate Cancer patients	
Ancestral group		African	European
Number of patients		113	57
Country of origin	South Africa	113	4
	Australia	0	53
Mean age		67 (45-99, Std dev = 8.4)[3]	62 (46-72, Std dev = 6.0)
Median preoperative PSA[1]		244 (4.3 – 4847)[4]	9.4 (3.5 – 31.8)
PSA>20 ng/mL*		65	40
PSA≤20 ng/mL		36	17
ISUP GG[2]	1	8	7
	2	10	0
	3	12	0
	4*	42	9
	5*	39	40
	unknown	2	1

[1]PSA: Prostate Specific Antigen (ng/mL).
[2]ISUP GG: International Society of Urological Pathology Group Grading.
[3]One patient in African ancestry has no record of age.
[4]Twelve patients in African ancestry have no record of exact PSA.
*NCCN inclusion criteria for high-risk/very high-risk PCa defined by ISUP GG/PSA

Supplementary Table 2. The count of germline SVs discovered and genotyped in each ancestral group.

SV filtering criteria	Population	Total	DEL	DUP	INS	INV	TRA
High-quality SV calls	African	38,668 (18,674)	22,423 (10,846)	2,155 (1,160)	11,797 (5,350)	1,189 (758)	1,104 (560)
	European	24,292 (4,298)	14,377 (2,800)	1,385 (390)	7,169 (722)	685 (254)	676 (132)
	All	42,966	25,223	2,545	12,519	1,443	1,236
High-quality genotype calls	African	28,515 (19,114)	16,903 (11,508)	1,451 (1,246)	8,350 (5,152)	1,033 (720)	778 (488)
	European	14,129 (4,728)	8,519 (3,124)	688 (483)	3,961 (763)	550 (237)	411 (121)
	All	33,243	20,027	1,934	9,113	1,270	899
Gene-disruptive and MAF≤5%	African	1,407 (1,314)	747 (711)	86 (84)	74 (68)	156 (151)	344 (300)
	European	543 (450)	294 (258)	50 (48)	20 (14)	44 (39)	135 (91)
	All	1,857	1,005	134	88	195	435

*Private SVs in bracket

Supplementary Table 3. Control population identified gene-disruptive SVs defined as PP-SV or impacting the same gene as PP-SV in PCa patients.

Genes	Gene impact type[1]	chrom1	pos1	chrom2	pos2	SV type	MAF in control population[2]
OCA2	pLoF	chr15	28,017,719	chr15	28,020,677	DEL	0.02
DNAH9 – OCA2	pLoF	chr17	11,694,612	chr15	27,913,931	TRA	0.29
BARD1	pLoF	chr2	214,726,966	chr2	214,727,028	DEL	0.12
BTBD7 - SLC2A5	pLoF	chr14	93,246,136	chr1	9,061,394	TRA	0.17

[1] Gene impact type based on gene annotation. pLoF: Potential loss-of-function. CG: Copy gain. IED: Intragenic Exon Duplication.
[2] Minor Allele Frequency (MAF) of SVs were estimated based on high-quality genotype calls only (detail in Methods).

Supplementary Table 4. The count of SVs with unknown classification in ClinVar or absent from dbVar scored by SV impact prediction tools.

Tool	Total SVs	DEL	DUP	INS	INV	TRA
StrVCTVRE	409	353	56	0	0	0
CADD-SV	1,180	976	124	80	0	0
POSTRE	1,590	969	122	0	97	402
PhenoSV	1,796	972	128	87	190	419
Scored by all three tools	398	343	55	0	0	0
StrVCTVRE Score ≥0.37	136	114	22	0	0	0
CADD-SV Score ≥10	194	164	16	14	0	0
POSTRE Score ≥0.8	264	38	7	0	17	201
PhenoSV score ≥0.5	640	179	30	2	82	347
PASS thresholds of any two tools*	291	107	16	0	11	157
PASS thresholds of all four tools	2	2	0	0	0	0

*Our study criteria to define pathogenic candidate SVs.

Supplementary Table 5. Criteria to predict and establish the potential pathogenicity of SVs

SV types	Criteria to define PP-SV candidates[1]				PP-SV candidate count		
	CADD-SV score ≥10	StrVCTVRE score ≥ 0.37	POSTRE score ≥ 0.8	PhenoSV score ≥ 0.5	African-private	European-private	Shared
DEL	✓	✓	✓	✓	67	39	1
DUP	✓	✓	✓	✓	7	9	0
INS	×	✓	×	✓	0	0	0
INV	×	×	✓	✓	9	2	0
TRA	×	×	✓	✓	107	38	12

[1]Potential Pathogenic candidate DEL or DUP were required to meet two of the criteria.

Supplementary Table 6. Pathogenic scores computed by SV impact prediction tools for SVs in ClinVar.

chrom	start	end	SV type	ClinVar Significance	StrVCTVRE score	CADD-SV score	POSTRE score	PhenoSV score
chr2	44281377	44281612	DUP	Likely pathogenic	**0.54**	**10.68**	0.75	**0.99**
chr15	28017719	28020677	DEL	Likely pathogenic	**0.58**	4.3	**0.88**	**0.56**
chr18	62152637	62157701	DEL	Pathogenic	**0.62**	7.5	0.67	**0.94**
chr1	231184145	231186249	DEL	Benign	NA	4.4	0.75	0.31
chr2	132488843	132490840	DEL	Benign	NA	4.8	0.15	0.03
chr3	130413180	130414060	DEL	Benign	**0.48**	7.0	0.75	0.39
chr5	177098012	177102222	DEL	Benign	0.07	**12.8**	0.63	0.02
chr9	6672237	6675374	DEL	Benign	NA	4.7	0.5	0.35
chr9	133252413	133258230	DEL	Benign	**0.44**	0.89	0.49	0.03
chr10	52766742	52769474	DEL	Benign	0.18	5.1	0	**0.53**
chr12	3461663	3463037	DEL	Benign	NA	3.5	0.33	**0.52**
chr15	33864126	33864212	DUP	Uncertain significance	**0.55**	9.6	0.33	**0.97**
chr17	73749723	73751769	DEL	Benign	NA	**17.1**	0.5	0.16
chr18	56089793	56090590	DEL	Benign	NA	4.3	0.63	0.20

*Scores in bold indicate meeting the criterion of individual SV impact prediction tool for pathogenicity as defined by our study.

Supplementary Table 7. Literature review on tumour suppressor or oncogenic effect of genes disrupted by cancer-related PP-SV candidates.

Gene name	SV type	Potential impact in cancer	Cancer type	Reference
ATAD2	DEL	Oncogenic effect	Multiple cancers	[1]
F5	DEL	Oncogenic effect	Gastric cancer and PCa	[2, 3]
HDAC9	DEL	Oncogenic effect	Multiple cancers	[4, 5]
FKBP9	DEL	Oncogenic effect	PCa and glioblastoma	[6, 7]
ADAM2	DEL	Oncogenic effect	Lung cancer	[8]
GGA2	DEL	Oncogenic effect	Lung cancer	[9]
SELP	DEL	Oncogenic effect	glioblastoma	[10]
OAS3	DEL	Oncogenic effect	Multiple cancers	[11]
CYRZ	DEL	Unknown	DNA methylation-driven gene in PCa	[12]
TULP2	DEL	Unknown	Breast cancer associated gene in Genome-wide association studies	[13]
BCKDHB	DEL	Unknown	Downregulation in breast cancer	[14]
SLC7A2	DEL	Tumour suppressor	Ovarian cancer and lung cancer	[15, 16]
DNAJC15	DEL	Tumour suppressor	Breast cancer	[17]
BCL2L11	DEL	Tumour suppressor	Gastric cancer	[18]
BARD1	DEL	DNA damage repair gene	Multiple cancers	[19]
COL4A2	DUP	Oncogenic effect	Gastric cancer and breast cancer	[20, 21]
BIRC6	DUP	Oncogenic effect	PCa, lung cancer and breast cancer	[22, 23, 24, 25]
LTBP1	DUP	Oncogenic effect	Lymphoma, PCa, Esophageal squamous cell carcinoma, breast cancer	[26, 27, 28, 29, 30]
		Tumour suppressor	Cervical caner	[30]
SLC2A5	DUP	Oncogenic effect	Multiple cancers	[31]
MLH1	INV	DNA mismatch repair gene	PCa and Lynch syndrome	[32, 33]
RB1	INV	Tumour suppressor	PCa	[34]
WASF1	INV	Tumour suppressor	PCa	[35]
FOXP1	INV	Tumour suppressor	PCa	[36, 37]

NSD3	INV	Oncogenic effect	Multiple cancers	[38, 39]
GRM8	TRA	Oncogenic effect	PCa	[40]
WDR43	TRA	Oncogenic effect	Colorectal cancer and lung cancer	[41, 42, 43]
NPM1	TRA	Oncogenic effect	PCa and lung cancer	[44, 45, 46]
NUSAP1	TRA	Oncogenic effect	PCa	[47]
MECOM	TRA	Oncogenic effect	Multiple cancers	[48, 49]
PKHD1	TRA	Oncogenic effect	Colon cancer	[50]
		Tumour suppressor	Colorectal cancer	[51]
CTNNA1	TRA	Tumour suppressor	Multiple cancers	[52]
PHC3	TRA	Tumour suppressor	PCa	[53]
PRKACA	TRA	Oncogenic effect	Adrenocortical cancer	[54]
KCTD3	TRA	Unknown	Mutation associated with neurogenetic and neurodevelopmental disorders	[55]
DST	TRA	Tumour suppressor	Breast cancer	[56]
AK8	TRA	Tumour suppressor	Uterine Carcinosarcoma	[57]

Supplementary Table 8. Variant allele frequency (VAF) measurements for PP-SVs

Gene name	SV type	chrom1	pos1	chrom2	pos2	Patient ID	Altered read count	Total read count[1]	VAF
SLC3A1	DUP	chr2	44281377	chr2	44281612	N0001	19.1	57.8	0.33
		chr2	44281377	chr2	44281612	SMU094	16.3	54.6	0.30
OCA2	DEL	chr15	28017719	chr15	28020677	N0059	15.5	34.2	0.45
PIGN	DEL	chr18	62152637	chr18	62157701	SMU083	17.4	34.2	0.51
SLC7A2	DEL	chr8	17418976	chr8	17544122	UP2035	21.0	43.4	0.48
		chr8	17418976	chr8	17544122	KAL0054	15.6	31.6	0.49
DNAJC15	DEL	chr13	43078470	chr13	43079390	17135	19.6	40.4	0.48
BCL2L11	DEL	chr2	111122626	chr2	111125901	KAL0101	17.8	39.7	0.45
BARD1	DEL	chr2	214768022	chr2	214772899	N0073	15.1	31.2	0.49
COL4A2/COL4A1	DUP	chr13	110294204	chr13	110633815	UP2039	20.5	58.6	0.35
SLC2A5	DUP	chr1	9045605	chr1	9049441	11099	23.7	73.8	0.33
FOXP1	INV	chr3	71097066	chr3	74525618	UP2101	20.3	44.4	0.41
		chr3	71097066	chr3	74525618	N0084	16.0	39.1	0.41
WASF1	INV	chr6	108167886	chr6	110172775	N0048	17.5	54.2	0.32
MLH1	INV	chr3	37000362	chr3	39352689	SMU080	13.5	31.2	0.43
RB1	INV	chr13	48466588	chr13	48473911	SMU064	12.0	32.3	0.37
CTNNA1	TRA	chr5	138903881	chr19	21614900	13179	19.5	38.6	0.50
AK8-DST	TRA	chr9	132876361	chr6	56896165	11452	17.7	37.9	0.47
LTBP1/BIRC6	DUP	chr2	32403832	chr2	33107415	5287	23.1	59.7	0.39
PHC3-PRKACA	TRA	chr3	170090742	chr19	14110142	SMU061	22.5	45.7	0.49
KCTD3-DST	TRA	chr1	215567414	chr6	56652607	UP2039	16.0	40.3	0.40
		chr1	215567414	chr6	56652607	SMU101	20.0	47.3	0.42
PKHD1	TRA	chr6	51981375	chr15	30874073	N0056	18.0	50.6	0.36
		chr6	51981375	chr15	30874073	SMU196	23.0	58.8	0.39

[1]For DEL, the total read count is the average read depth of 10 kbp upstream and downstream of DEL region. For DUP, total read count is the average read depth of DUP region. For INV and TRA, total read count is defined as average read depth of $\pm$150 bp region to breakpoints.

Supplementary Table 9. Second somatic hit in tumour-matched samples from PP-SV presenting patients.

**chrom**	**start**	**end**	**gene**	**Patient ID**	**GISTIC predicted copy number value**
chr2	43470330	44640339	*SLC3A1*	SMU094	-1
chr15	27,017,069	27,907,116	*OCA2*	N0059	2
chr8	17,424,247	17,544,245	*SLC7A2*	UP2035	-1
chr3	70,951,922	72,671,968	*FOXP1*	UP2101	1
chr6	109,780,912	110,170,915	*WASF1*	N0048	2
chr2	32,400,246	33,110,251	*LTBP1*, *BIRC6*	5287	2
chr1	215,538,501	215,618,505	*KCTD3*	UP2039	-1
chr6	56,313,745	56,623,766	*DST*	UP2039	-1
chr6	51,563,429	52,363,482	*PKHD1*	N0056	1

Supplementary Table 10. Sequencing depth of coverage in identified potentially pathogenic DEL or DUP regions.

**Gene name**	**chrom1**	**pos1**	**chrom2**	**pos2**	**SV type**	**Sample**	**Average depth in SV region (X)**	**Average depth in 10 kb upstream of SV region (X)**	**Average depth in 10 kb downstream of SV region (X)**
SLC3A1	chr2	44281377	chr2	44281612	DUP	N0001	57.8	39.0	38.5
						SMU094	54.6	38.6	37.9
OCA2	chr15	28017719	chr15	28020677	DEL	N0059	18.7	33.6	34.9
PIGN	chr18	62152637	chr18	62157701	DEL	SMU083	16.7	33.9	34.3
SLC7A2	chr8	17418976	chr8	17544122	DEL	UP2035	22.4	43.6	43.2
						KAL0054	16.0	32.3	30.9
DNAJC15	chr13	43078470	chr13	43079390	DEL	17135	20.8	41.2	39.5
BCL2L11	chr2	111122626	chr2	111125901	DEL	KAL0101	21.9	43.8	35.6
BARD1	chr2	214768022	chr2	214772899	DEL	N0073	16.1	30.9	31.5
COL4A2/ COL4A1	chr13	110294204	chr13	110633815	DUP	UP2039	58.6	37.7	38.5
SLC2A5	chr1	9045605	chr1	9049441	DUP	11099	73.8	51.0	48.9
LTBP1/ BIRC6	chr2	32403832	chr2	33107415	DUP	5287	59.7	35.3	38.0

Supplementary Figures

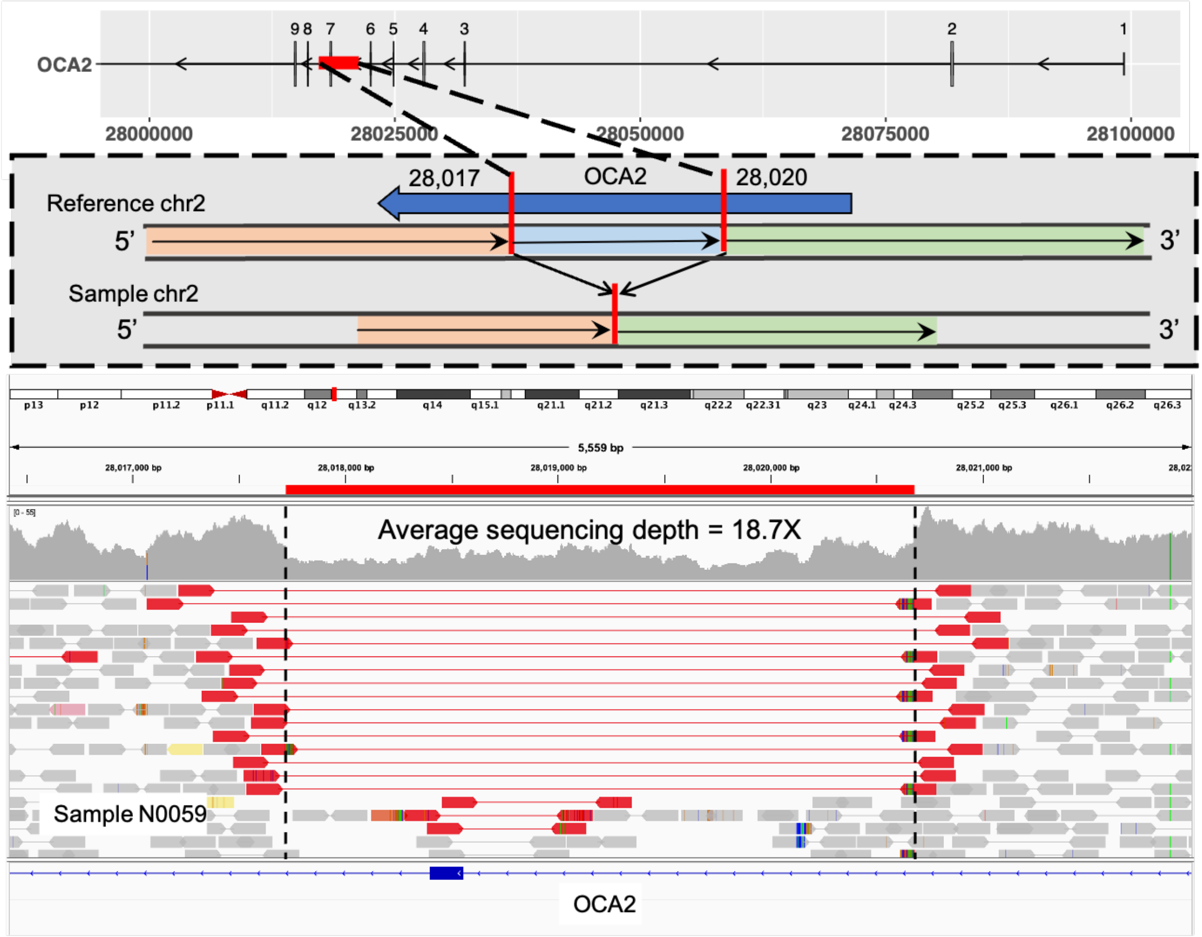

Supplementary Figure 1. *OCA2* pLoF deletion reported as "likely pathogenic" in ClinVar. Top row shows transcript structure of *OCA2* and deletion region highlighted in red. Middle row shows the DEL schematic diagram. Bottom row shows the sequencing read depth and alignments in DEL region using Integrative Genomic Viewer. Both the discordantly aligned read-pairs (red) and split-reads (soft-clipped bases in rainbow colour) support the existence of the DEL and its breakpoints. DEL breakpoints are shown in dashed black vertical lines.

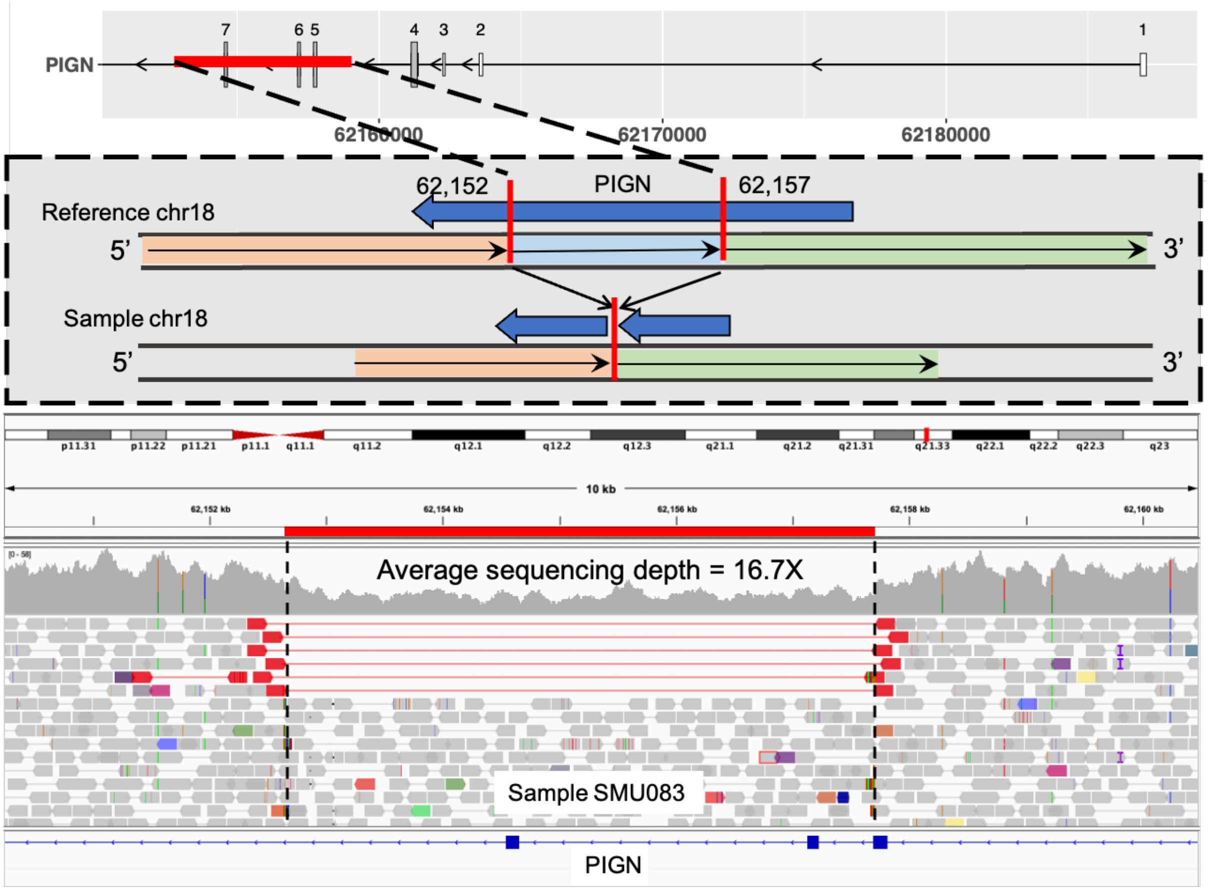

Supplementary Figure 2. *PIGN* pLoF deletion reported as “pathogenic” in ClinVar. Top row shows transcript structure of *PIGN* and deletion region highlighted in red. Middle row shows the DEL schematic diagram. Bottom row shows the sequencing read depth and alignments in DEL region using Integrative Genomic Viewer. Both the discordantly aligned read-pairs (red) and split-reads (soft-clipped bases in rainbow colour) support the existence of the DEL and its breakpoints. DEL breakpoints are shown in dashed black vertical lines.

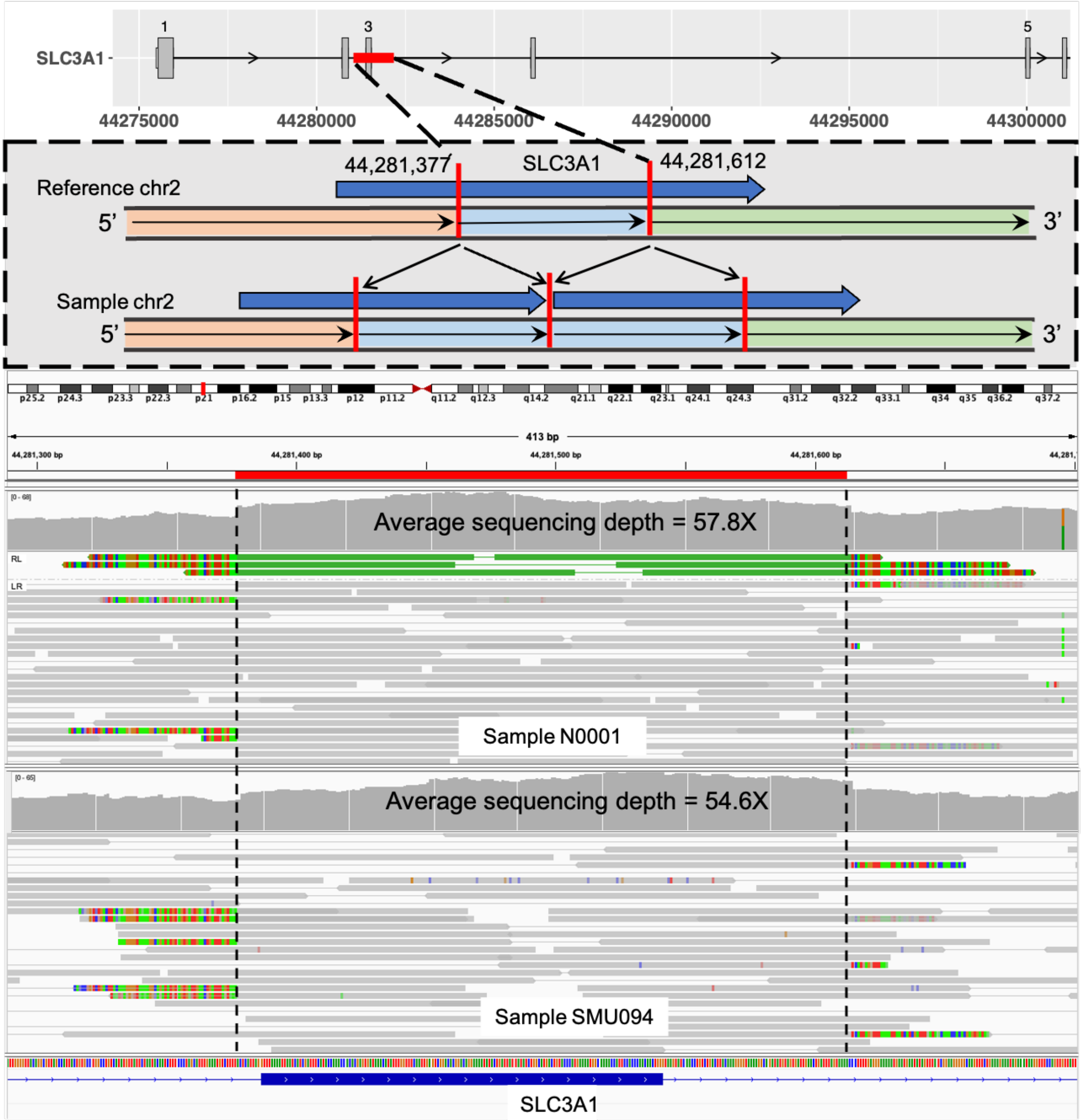

Supplementary Figure 3. ***SLC3A1*** **intragenic exon duplication reported as "likely pathogenic" in ClinVar.** Top row shows transcript structure of *SLC3A1* and DUP region highlighted in blue. Bottom row shows DUP supporting read-pairs (green) by visual inspection of aligned sequencing data using Integrative Genomic Viewer. DUP supporting read-pairs were not observed for sample SMU094, while higher coverage in the DUP region was observed.

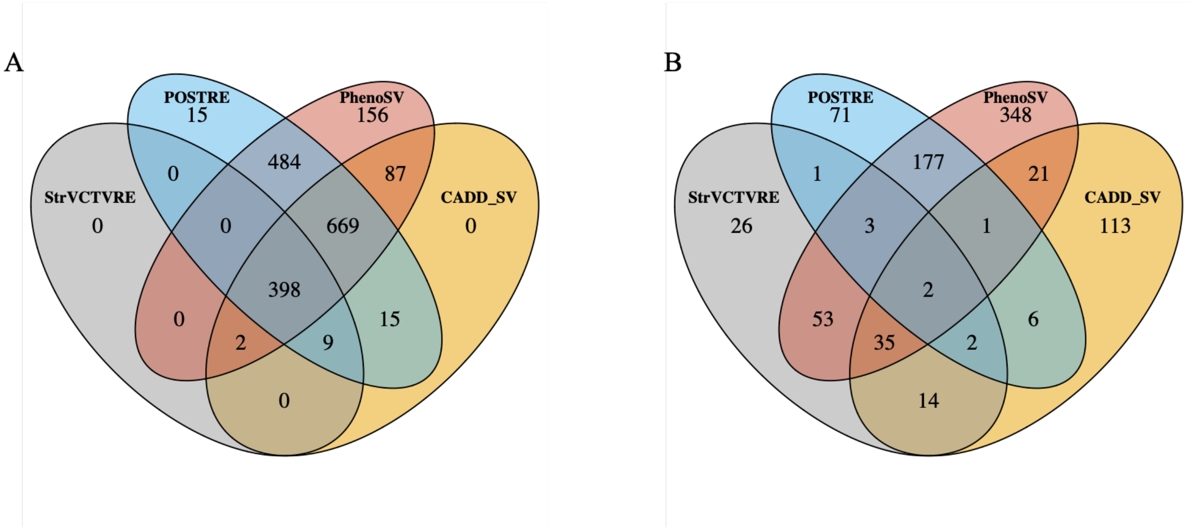

Supplementary Figure 4. Low-frequency gene-disruptive SVs with unknown classification in ClinVar or absent from dbVar scored by SV impact prediction tools. (A) Count of all scored SVs. (B) Count of SVs with CADD-SV score ≥ 10, StrVCTVRE score ≥ 0.37, POSTRE score ≥ 0.8 or PhenoSV score ≥ 0.5. Source data are provided as a Source Data file.

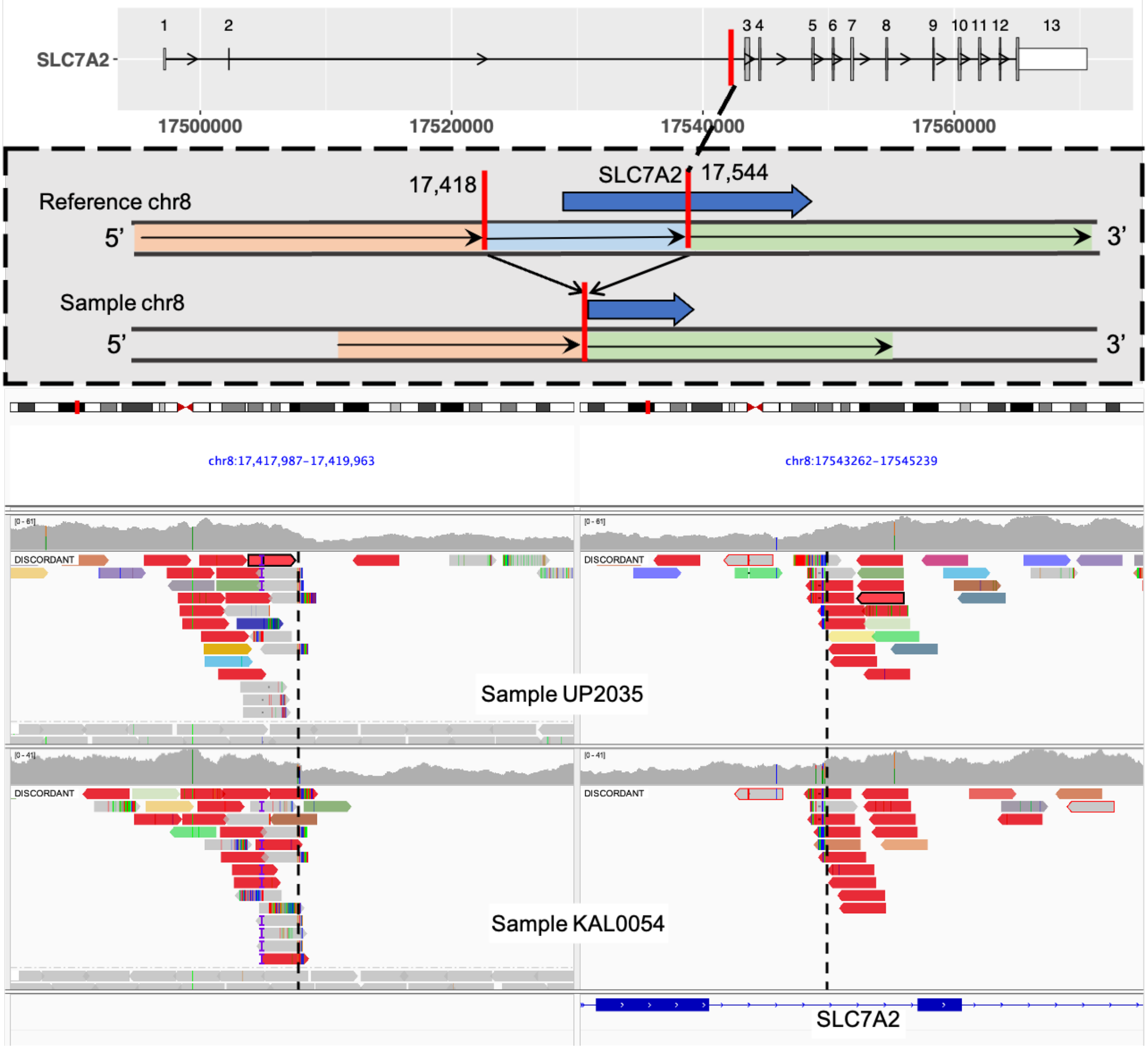

Supplementary Figure 5. ***SLC7A2*** **pLoF deletion identified as potentially pathogenic SV.** Top row shows transcript structure of *SLC7A2* and DEL breakpoint in red. Middle row shows the DEL schematic diagram. Bottom row shows the sequencing read depth and alignments around DEL breakpoints using Integrative Genomic Viewer. Both the discordantly aligned read-pairs (red) and split-reads (soft-clipped bases in rainbow colour) support the existence of the DEL and its breakpoints. DEL breakpoints are shown in dashed black vertical lines.

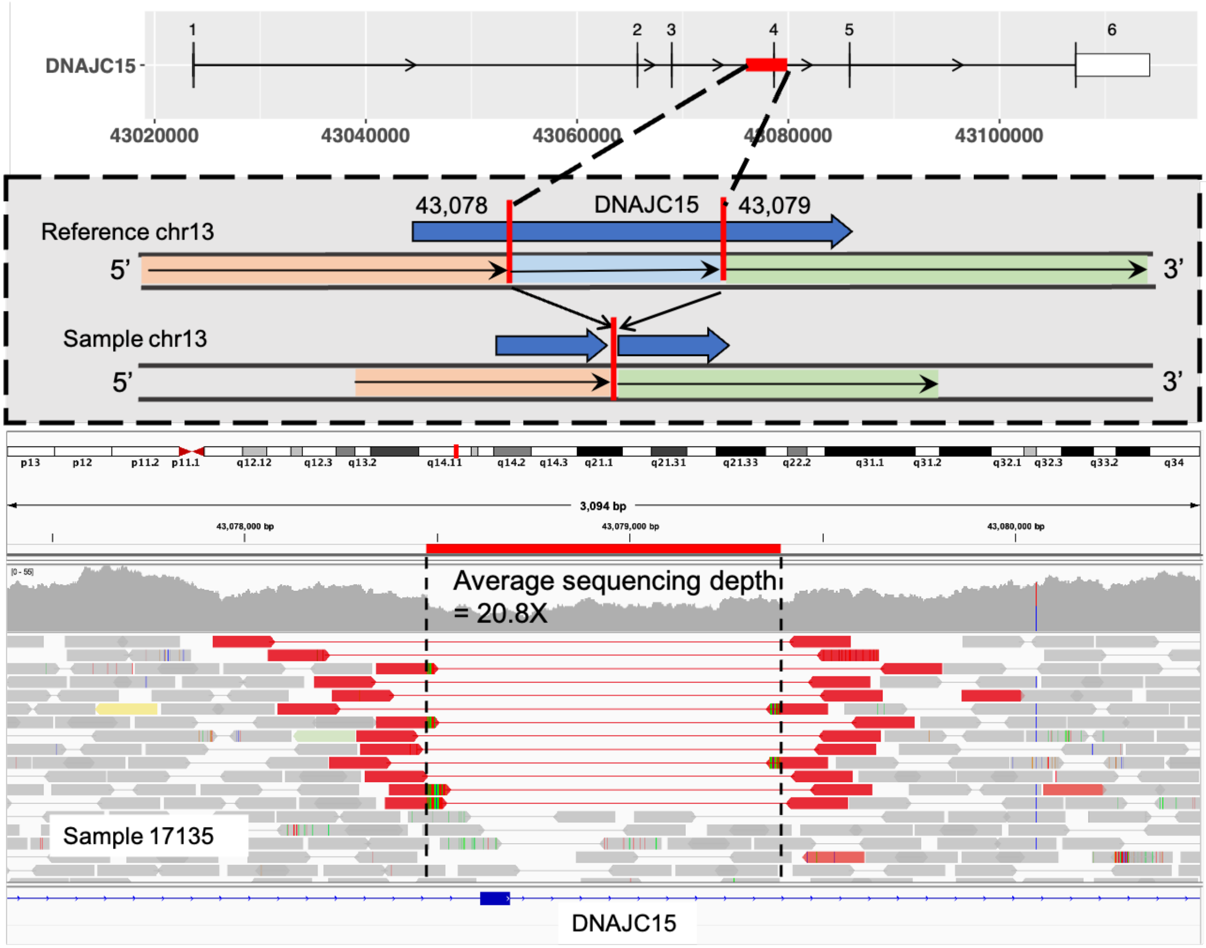

Supplementary Figure 6. *DNAJC15* pLoF deletion identified as potentially pathogenic SV. Top row shows transcript structure of *DNAJC15* with DEL region highlighted in read. Middle row shows the DEL schematic diagram. Bottom row shows the sequencing read depth and alignments in DEL region using Integrative Genomic Viewer. Both the discordantly aligned read-pairs (red) and split-reads (soft-clipped bases in rainbow colour) support the existence of the DEL and its breakpoints. DEL breakpoints are shown in dashed black vertical lines.

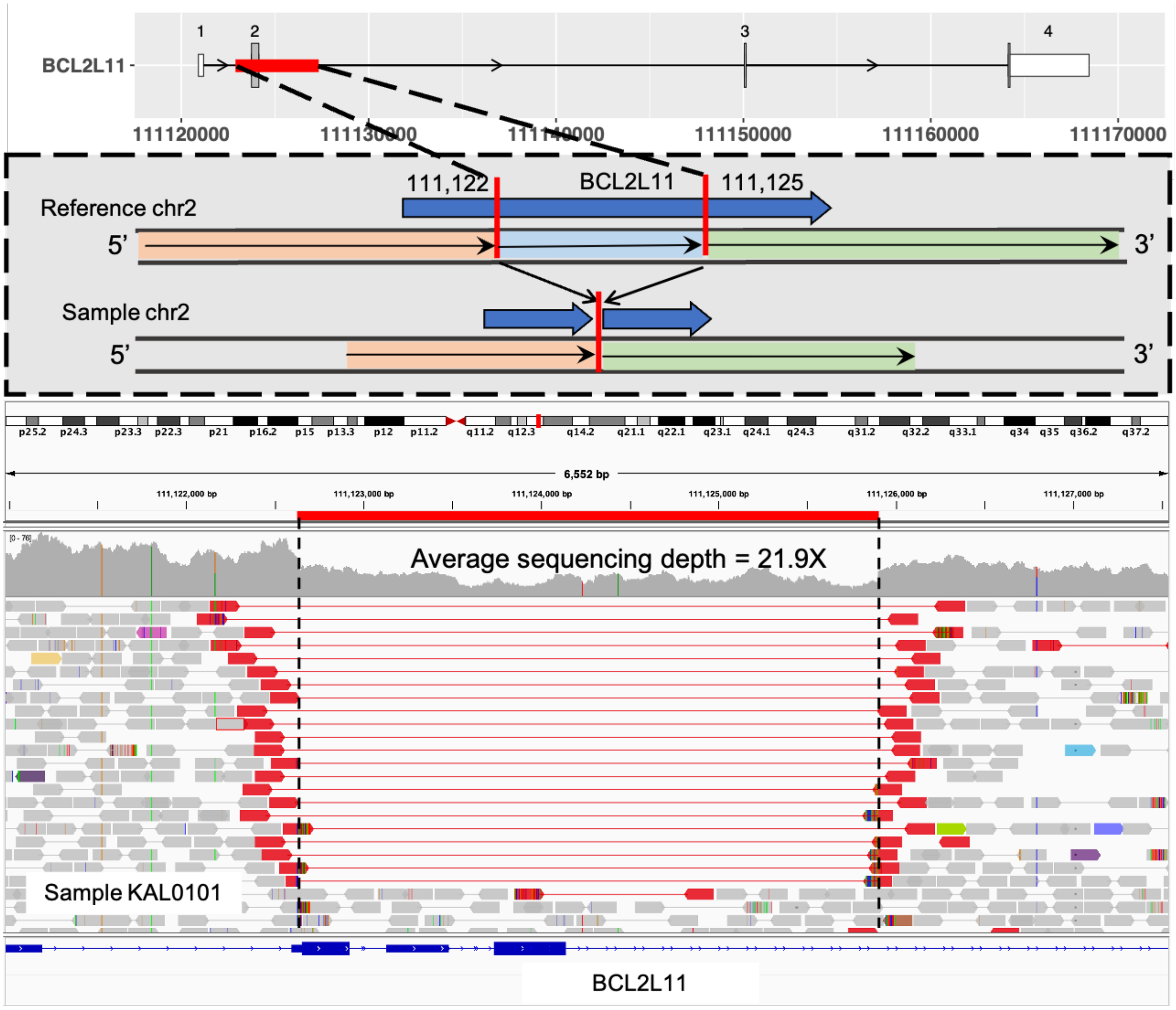

Supplementary Figure 7. ***BCL2L11*** **pLoF deletion identified as potentially pathogenic SV.** Top row shows transcript structure of *BCL2L11* with DEL region highlighted in read. Middle row shows the DEL schematic diagram. Bottom row shows the sequencing read depth and alignments in DEL region using Integrative Genomic Viewer. Both the discordantly aligned read-pairs (red) and split-reads (soft-clipped bases in rainbow colour) support the existence of the DEL and its breakpoints. DEL breakpoints are shown in dashed black vertical lines.

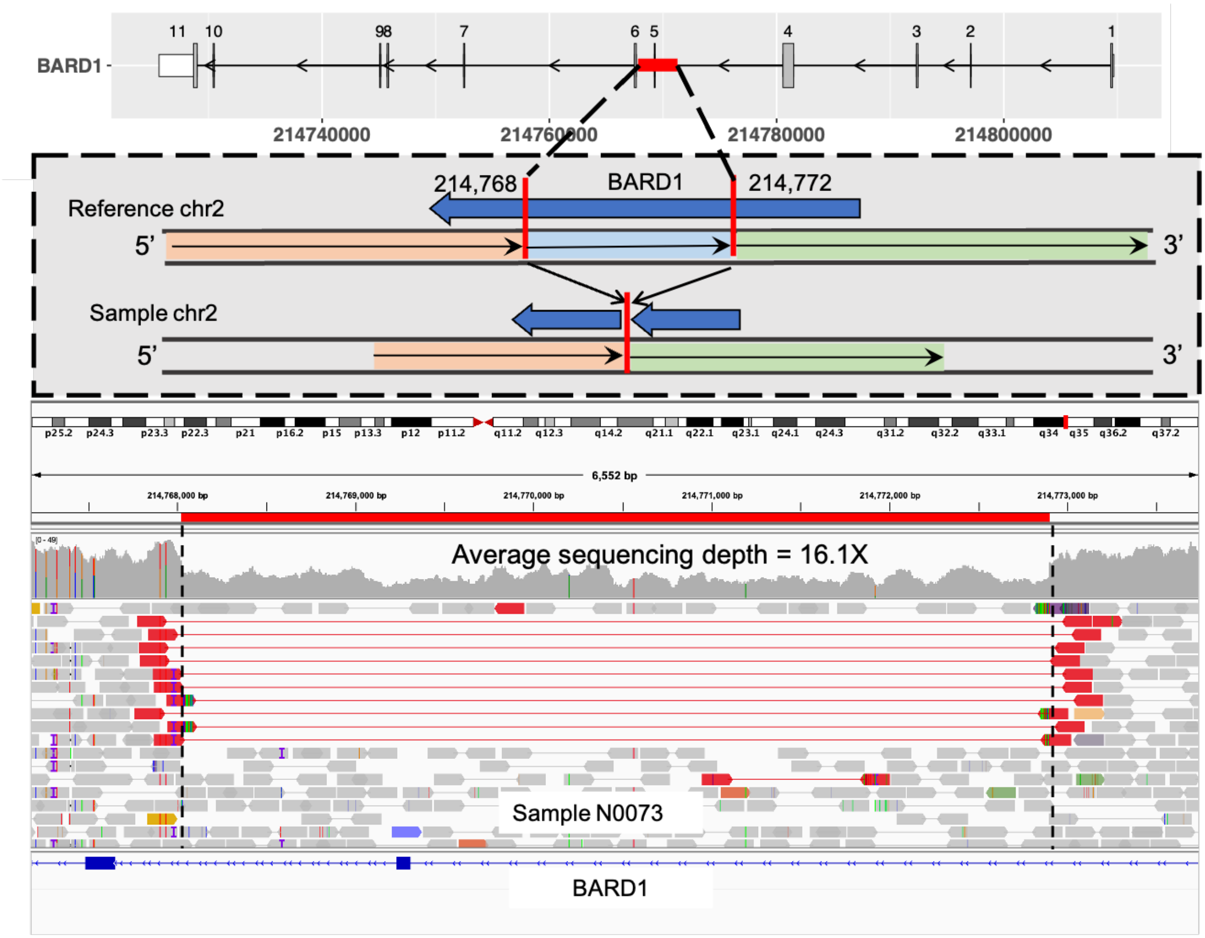

Supplementary Figure 8. *BARD1* pLoF deletion identified as potentially pathogenic SV. Top row shows transcript structure of *BARD1* with DEL region highlighted in read. Middle row shows the DEL schematic diagram. Bottom row shows the sequencing read depth and alignments in DEL region using Integrative Genomic Viewer. Both the discordantly aligned read-pairs (red) and split-reads (soft-clipped bases in rainbow colour) support the existence of the DEL and its breakpoints. DEL breakpoints are shown in dashed black vertical lines.

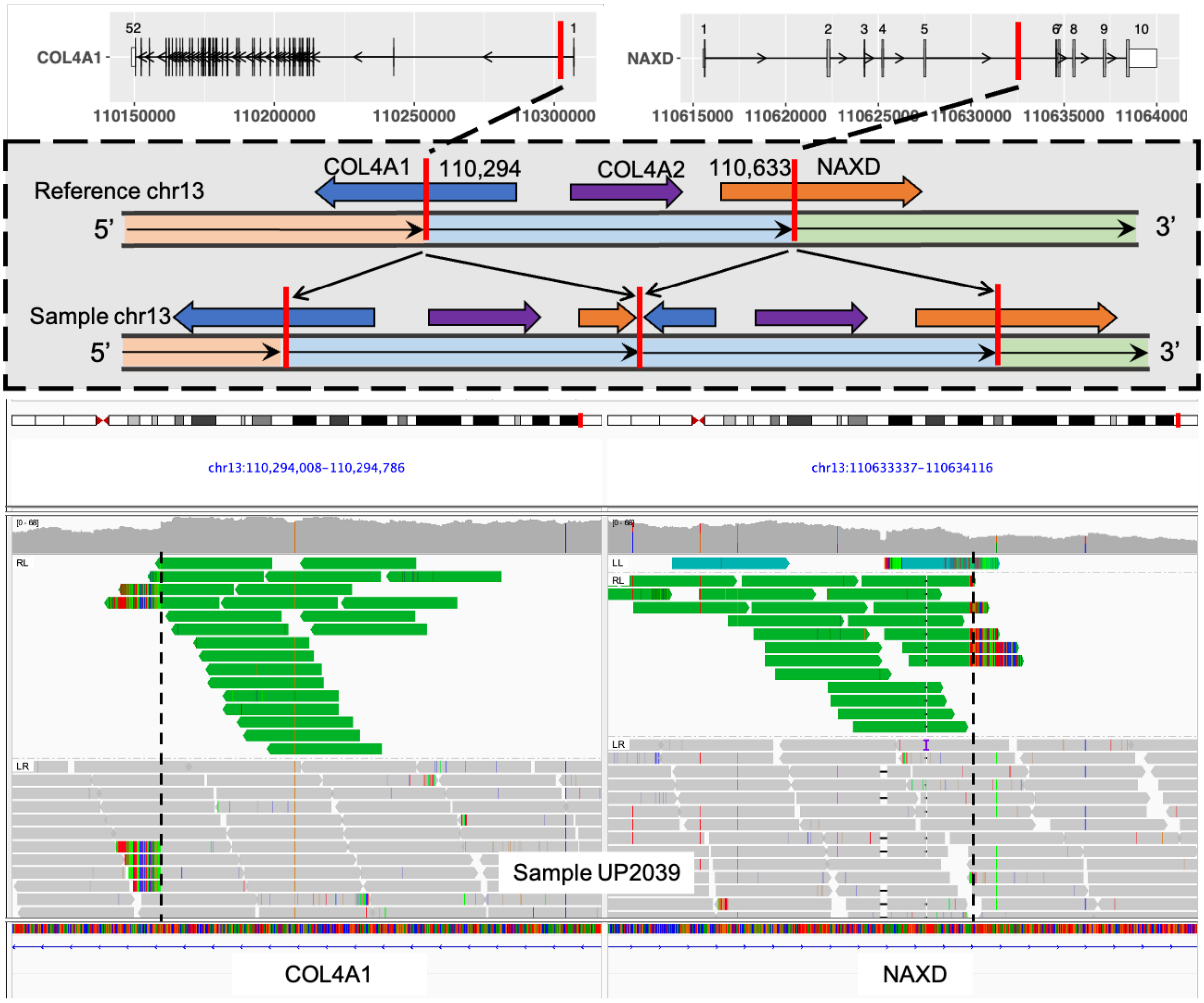

Supplementary Figure 9. ***COL4A2*** **whole-gene DUP identified as potentially pathogenic SV.** This dbVar reported 339,611 base DUP with breakpoints disrupting *COL4A1* and *NAXD*. Top row shows transcript structure of *COL4A1* and *NAXD*, and DUP breakpoints in red. Middle row shows the DUP schematic diagram. Bottom row shows the sequencing read depth and alignments around DUP breakpoints using Integrative Genomic Viewer. Both the discordantly aligned read-pairs (green) and split-reads (soft-clipped bases in rainbow colour) support the existence of the DUP and its breakpoints. DUP breakpoints are shown in dashed black vertical lines.

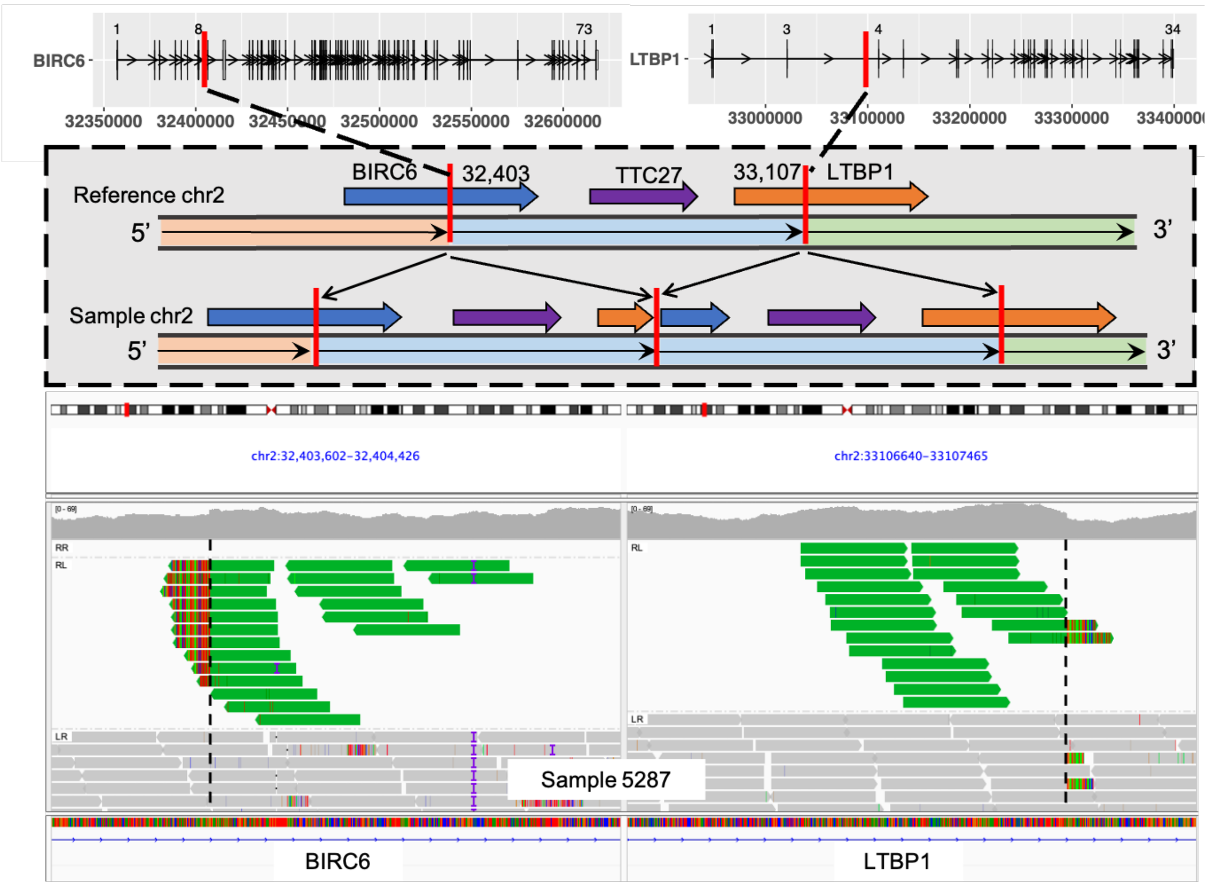

Supplementary Figure 10. Whole-gene DUP identified as "cautionary" potentially pathogenic SV. This dbVar reported 703,583 base DUP with breakpoints disrupting *LTBP1* and *BIRC6*. Top row shows transcript structure of *LTBP1* and *BIRC6*, and DUP breakpoints in red. Middle row shows the DUP schematic diagram. Bottom row shows the sequencing read depth and alignments around DUP breakpoints using Integrative Genomic Viewer. Both the discordantly aligned read-pairs (green) and split-reads (soft-clipped bases in rainbow colour) support the existence of the DUP and its breakpoints. DUP breakpoints are shown in dashed black vertical lines.

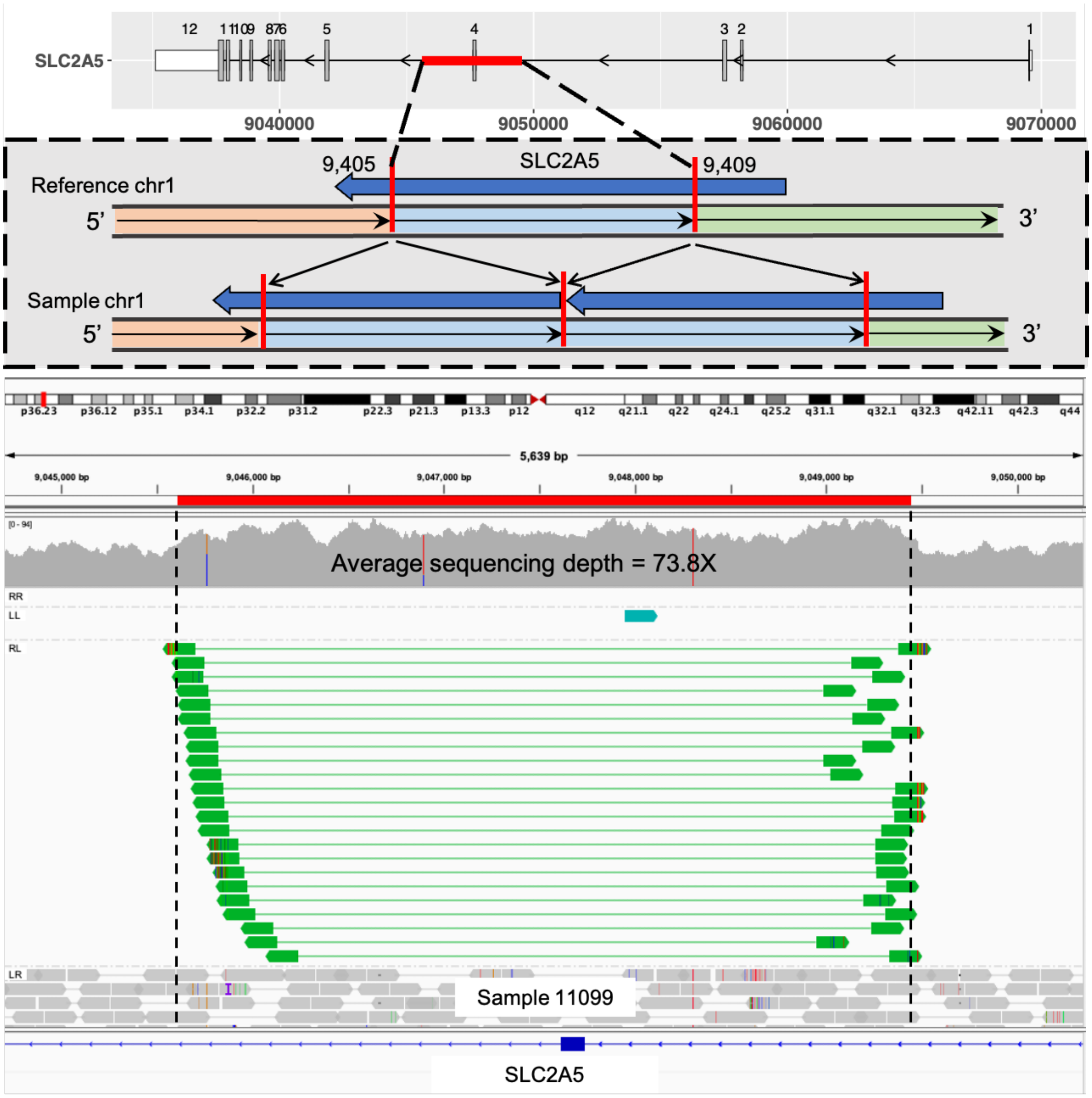

Supplementary Figure 11. ***SLC2A5*** **intragenic exon DUP identified as potentially pathogenic SV.** Top row shows transcript structure of *SLC2A5*, with DUP region in red. Middle row shows the DUP schematic diagram. Bottom row shows the sequencing read depth and alignments in DUP region using Integrative Genomic Viewer. Both the discordantly aligned read-pairs (green) and split-reads (soft-clipped bases in rainbow colour) support the existence of the DUP and its breakpoints. DUP breakpoints are shown in dashed black vertical lines.

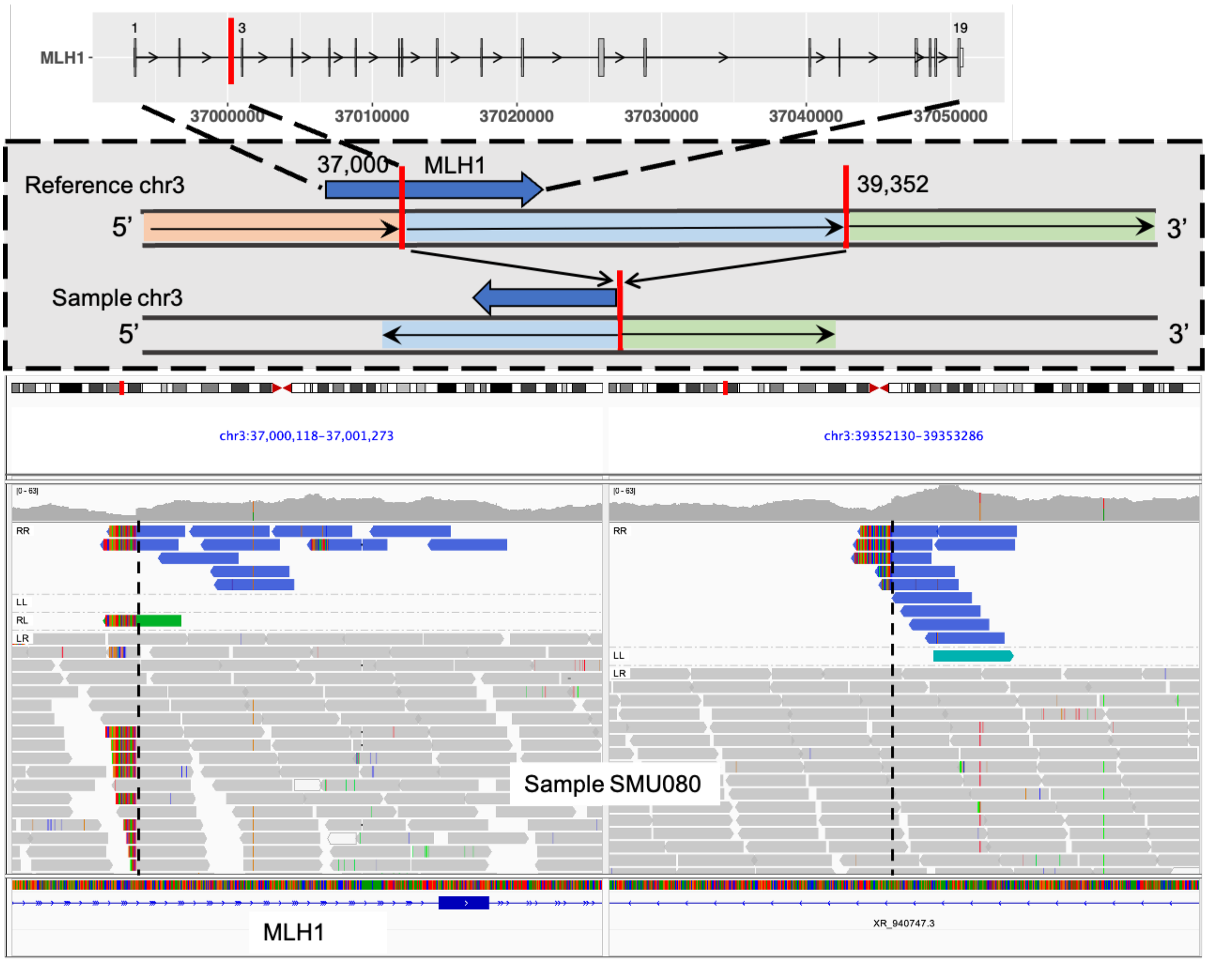

Supplementary Figure 12. *MLH1* pLoF inversion identified as potentially pathogenic SV. Top row shows transcript structure of *MLH1* and INV breakpoint in red. Middle row shows the INV schematic diagram. Bottom row shows the sequencing read depth and alignments around INV breakpoints using Integrative Genomic Viewer. Both the discordantly aligned read-pairs (blue) and split-reads (soft-clipped bases in rainbow colour) show the existence of the INV and its breakpoints. INV breakpoints are shown in dashed black vertical lines.

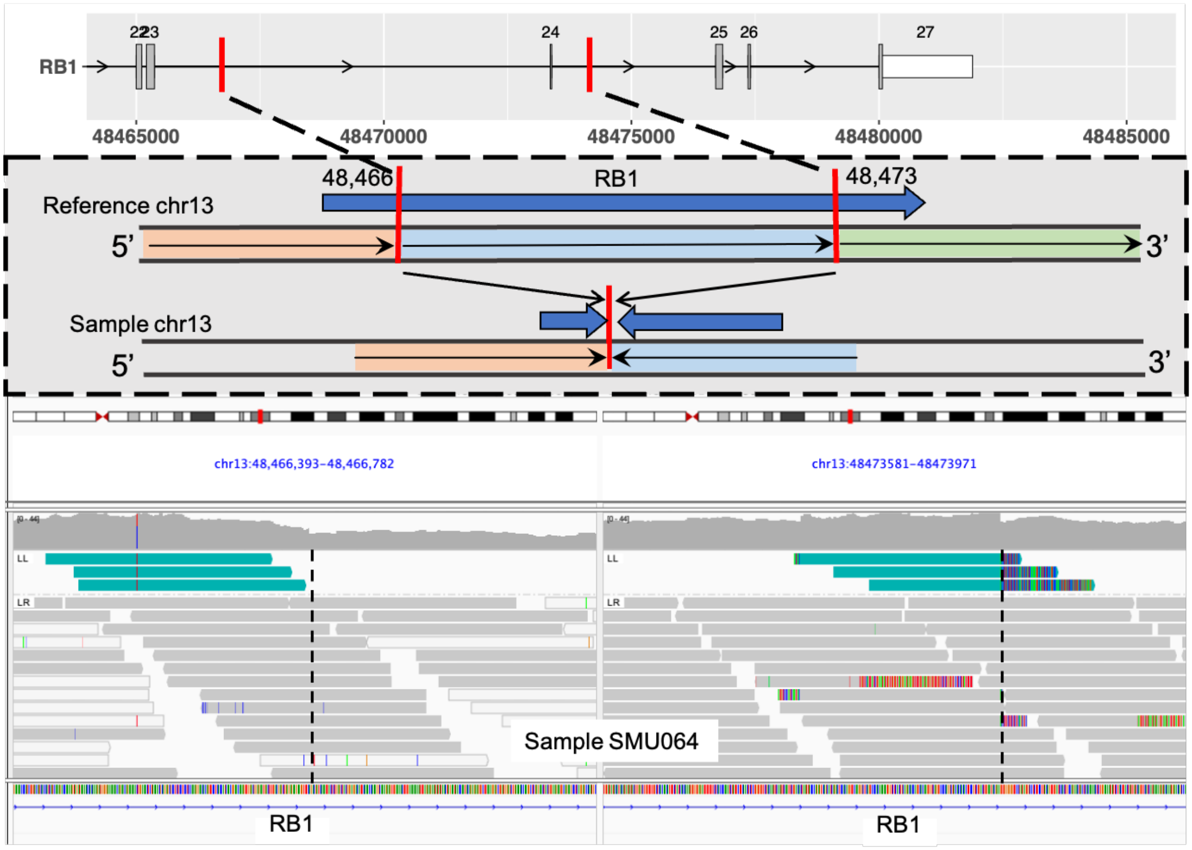

Supplementary Figure 13. *RB1* pLoF inversion identified as potentially pathogenic SV. Top row shows transcript structure of *RB1* and INV breakpoint in red. Middle row shows the INV schematic diagram. Bottom row shows the sequencing read depth and alignments around INV breakpoints using Integrative Genomic Viewer. Both the discordantly aligned read-pairs (cyan) and split-reads (soft-clipped bases in rainbow colour) show the existence of the INV and its breakpoints. INV breakpoints are shown in dashed black vertical lines.

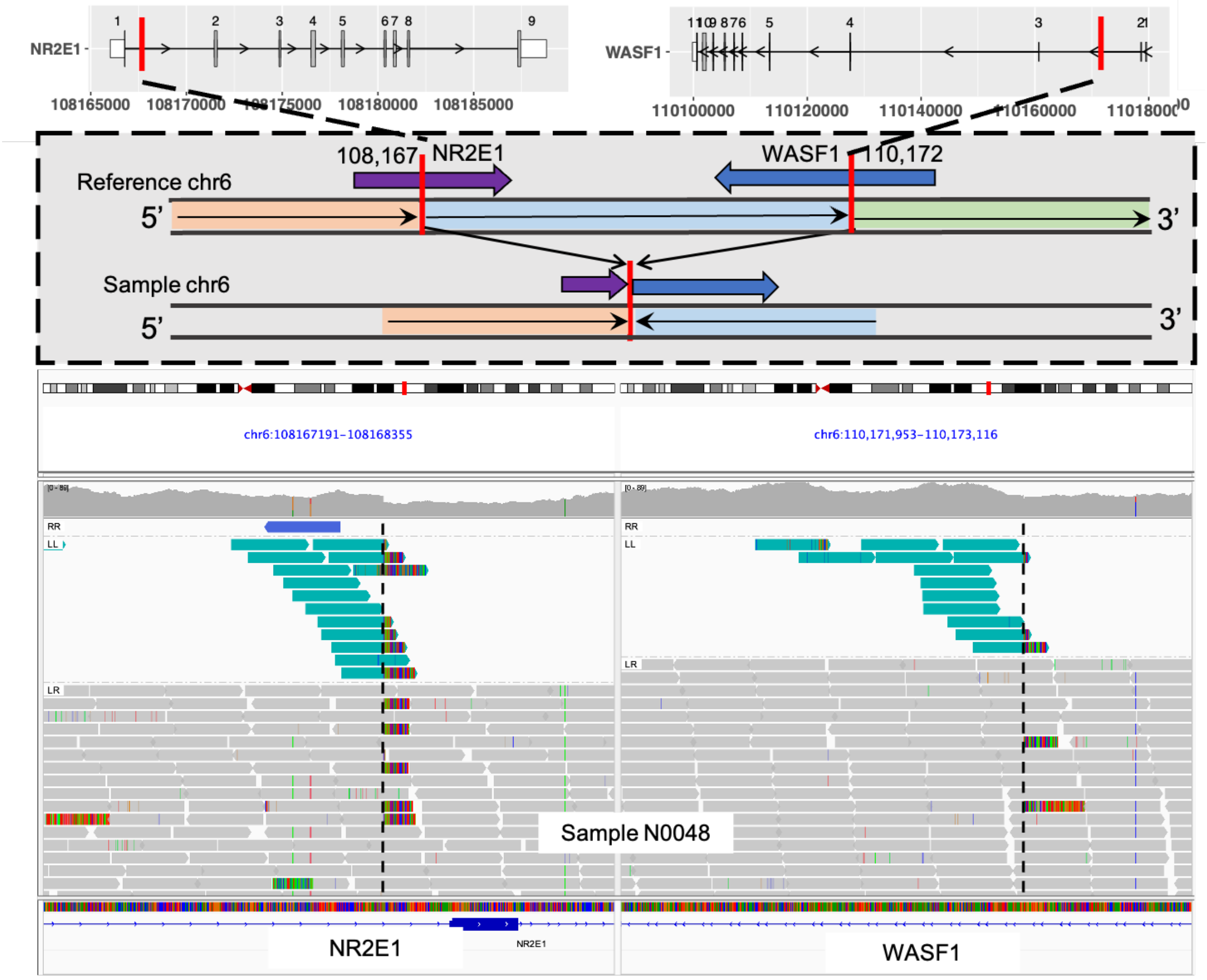

Supplementary Figure 14. *WASF1* pLoF inversion identified as potentially pathogenic SV. Top row shows transcript structure of *WASF1* and *NR2E1* and INV breakpoint in red. Middle row shows the INV schematic diagram. Bottom row shows the sequencing read depth and alignments around INV breakpoints using Integrative Genomic Viewer. Both the discordantly aligned read-pairs (cyan) and split-reads (soft-clipped bases in rainbow colour) show the existence of the INV and its breakpoints. INV breakpoints are shown in dashed black vertical lines.

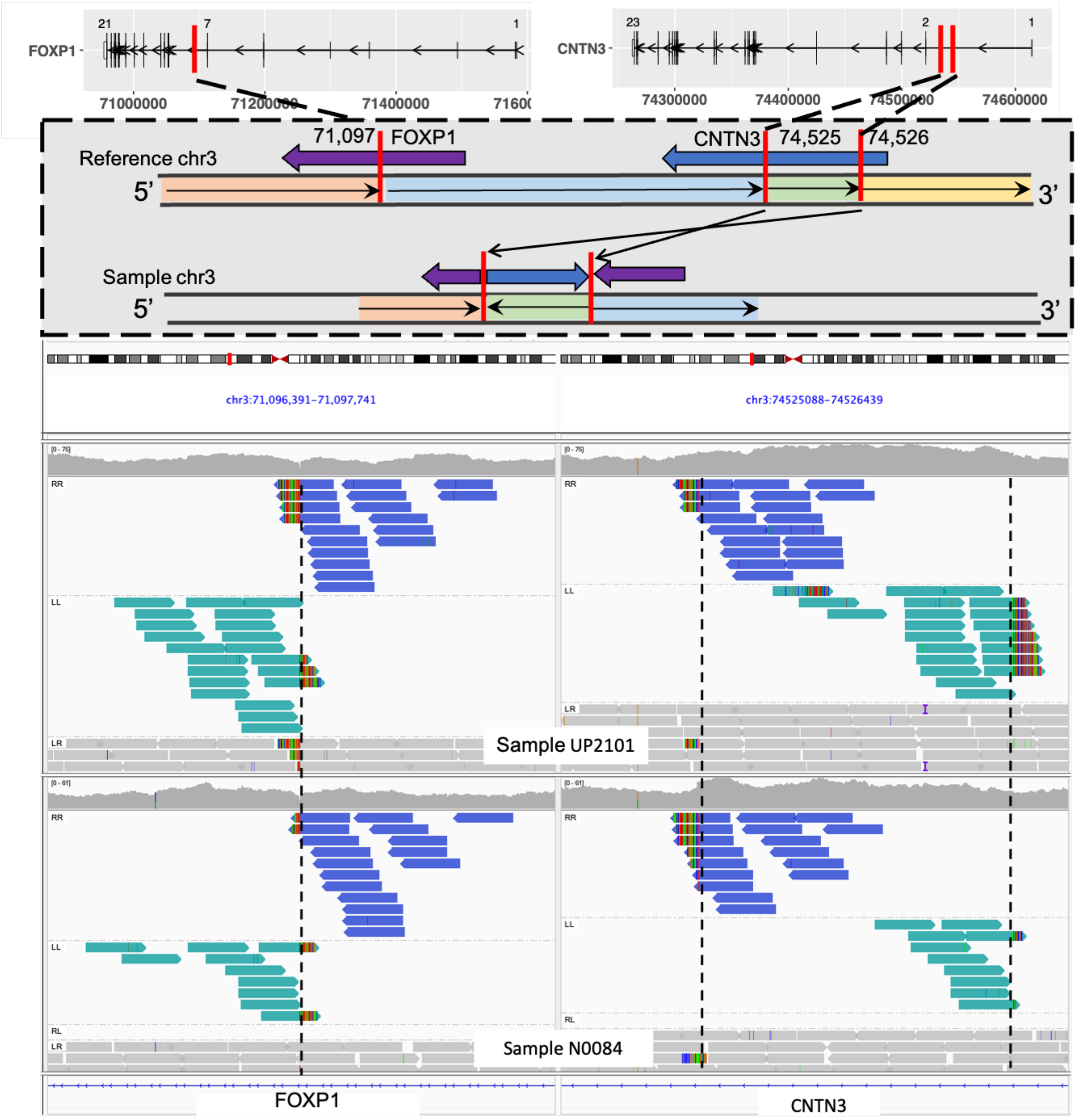

Supplementary Figure 15. *FOXP1* pLoF inversion identified as potentially pathogenic SV. Top row shows transcript structure of *FOXP1* and *CNTN3* and INV breakpoint in red. Middle row shows the INV schematic diagram. Bottom row shows the sequencing read depth and alignments around INV breakpoints using Integrative Genomic Viewer. Both the discordantly aligned read-pairs (blue and cyan) and split-reads (soft-clipped bases in rainbow colour) support the existence of the INV and its breakpoints. INV breakpoints are shown in dashed black vertical lines.

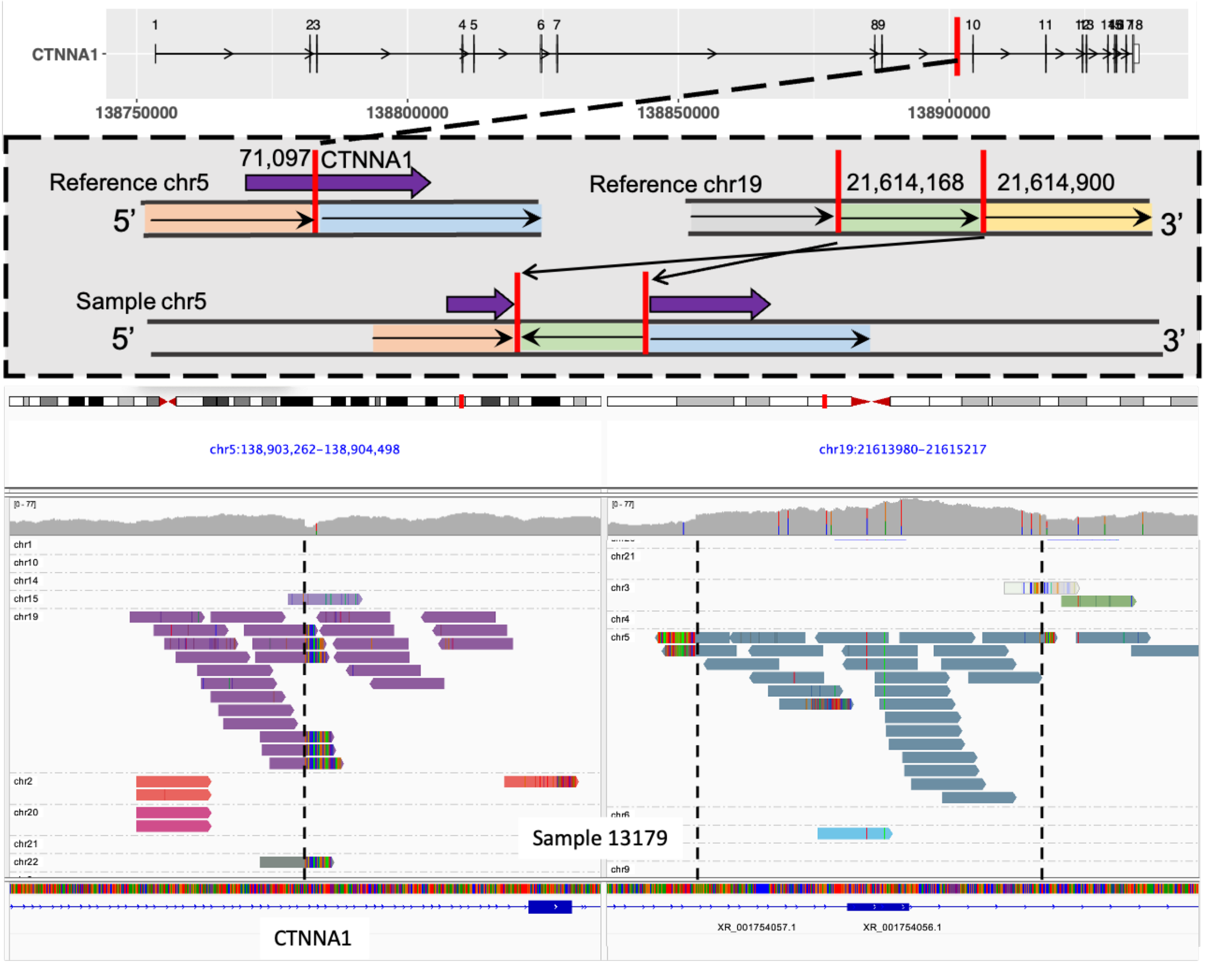

Supplementary Figure 16. *CTNNA1* translocation identified as potentially pathogenic SV. Top row shows transcript structure of *CTNNA1* and TRA breakpoint in red. Middle row shows the TRA schematic diagram. Bottom row shows the sequencing read depth and read alignments around TRA breakpoints using Integrative Genomic Viewer. Both the discordantly aligned read-pairs (purple and grey) and split-reads (soft-clipped bases in rainbow colour) support the existence of the TRA and its breakpoints. Sequencing reads with mate aligned to chr19 are in purple and reads with mate aligned to chr5 are in grey. TRA breakpoints are shown in dashed black vertical lines.

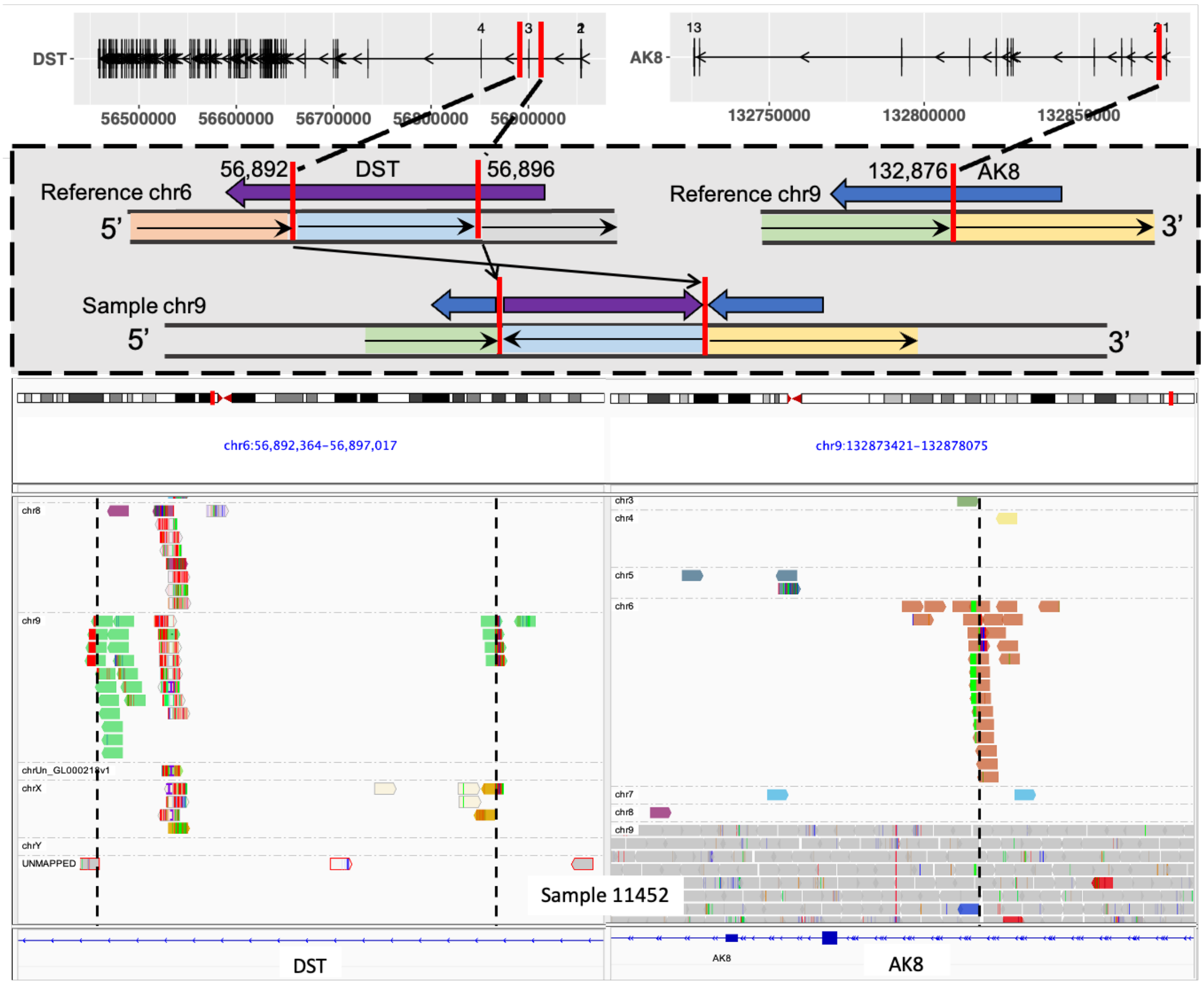

Supplementary Figure 17. *AK8-DST* Translocation identified as potentially pathogenic SV. Top row shows transcript structure of *DST* and *AK8* with TRA breakpoint in red. Middle row shows the TRA schematic diagram. Bottom row shows the sequencing read depth and read alignments around TRA breakpoints using Integrative Genomic Viewer. Both the discordantly aligned read-pairs (green and orange) and split-reads (soft-clipped bases in rainbow colour) support the existence of the TRA and its breakpoints. Sequencing reads with mate aligned to chr9 are in green and reads with mate aligned to chr6 are in orange. TRA breakpoints are shown in dashed black vertical lines.

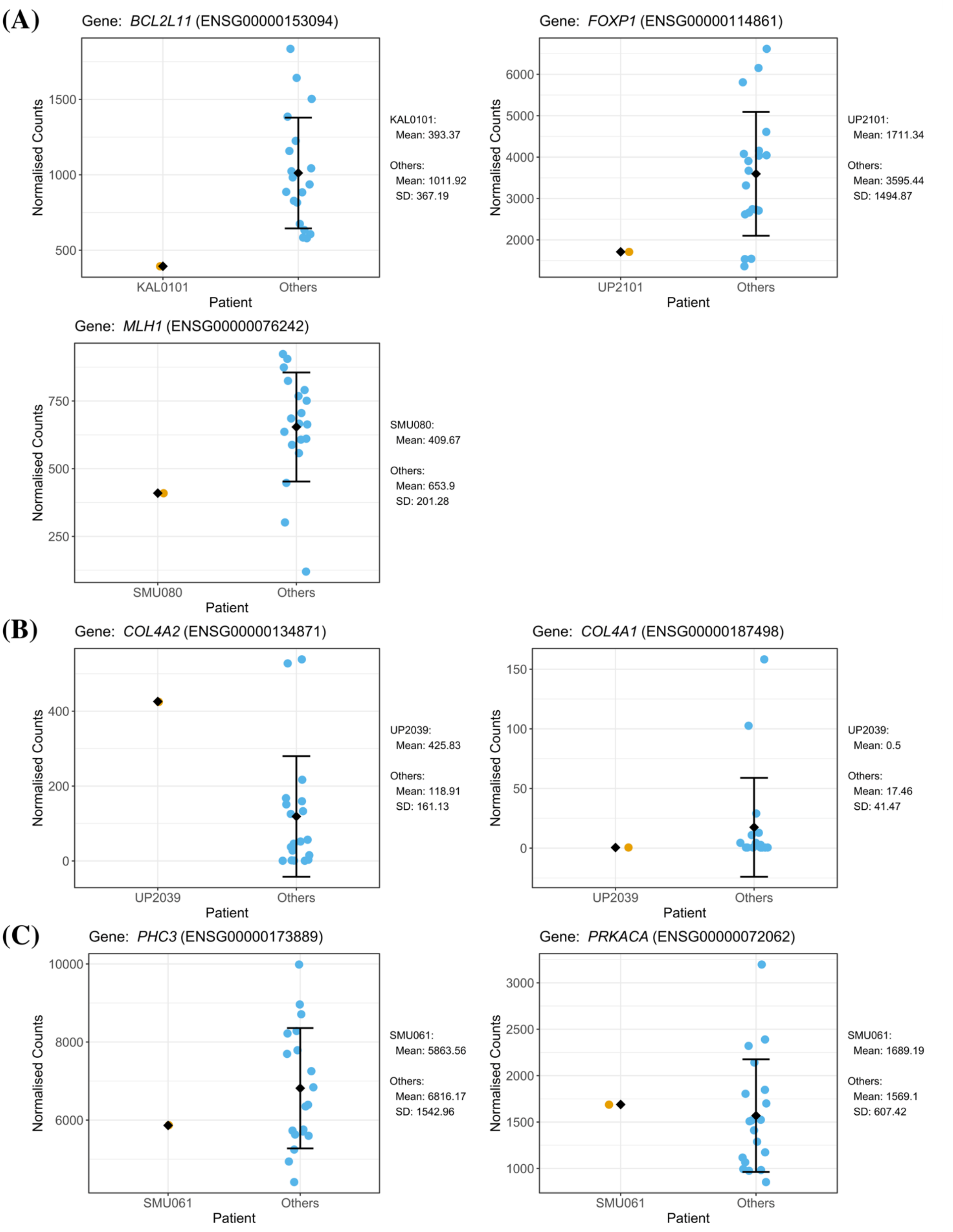

Supplementary Figure 18. Differential expression analysis of seven genes associated with potentially pathogenic SVs in the blood of 20 prostate cancer patients. (A) PP-SVs residing in *BCL2L11*, *FOXP1*, and *MLH1* in patients KAL0101, UP2101, and SMU080, respectively, are associated with the downregulation of the corresponding genes compared to other samples. (B) Patient UP2039 exhibited a high expression of *COL4A2*, while *COL4A1* is not expressed. (C) In patient SMU061, PP-SV affecting *PH3* and *PRKACA* shows no significant difference in expression levels to the cohort. Source data are provided as a Source Data file.

REFERENCES

1. Liu H*, et al.* Tumor-Promoting ATAD2 and Its Preclinical Challenges. *Biomolecules* **12**, (2022).

2. Liu Y, Liao XW, Qin YZ, Mo XW, Luo SS. Identification of F5 as a Prognostic Biomarker in Patients with Gastric Cancer. *Biomed Res Int* **2020**, 9280841 (2020).

3. Klee EW*, et al.* Candidate serum biomarkers for prostate adenocarcinoma identified by mRNA differences in prostate tissue and verified with protein measurements in tissue and blood. *Clin Chem* **58**, 599-609 (2012).

4. Patra R, Chakraborty J, Das NC, Mukherjee S. An integrated omics study on the role of HDAC9 gene in the oncogenic events of human gastrointestinal-tract associated cancers. *Human Gene* **37**, 201189 (2023).

5. Kim B, Sohn HM, Hyun H, Lim W. Effect of HDAC9 inhibition on epithelial-mesenchymal transition in CD133+ prostate cancer cell lines. *J Chemother* **34**, 45-54 (2022).

6. Jiang FN*, et al.* Increasing of FKBP9 can predict poor prognosis in patients with prostate cancer. *Pathol Res Pract* **216**, 152732 (2020).

7. Xu H*, et al.* FKBP9 promotes the malignant behavior of glioblastoma cells and confers resistance to endoplasmic reticulum stress inducers. *Journal of Experimental & Clinical Cancer Research* **39**, 44 (2020).

8. Dervovic D*, et al.* In vivo CRISPR screens reveal Serpinb9 and Adam2 as regulators of immune therapy response in lung cancer. *Nature Communications* **14**, 3150 (2023).

9. O'Farrell H*, et al.* Integrative Genomic Analyses Identifies GGA2 as a Cooperative Driver of EGFR-Mediated Lung Tumorigenesis. *J Thorac Oncol* **14**, 656-671 (2019).

10. Yeini E*, et al.* P-selectin axis plays a key role in microglia immunophenotype and glioblastoma progression. *Nature Communications* **12**, 1912 (2021).

11. Li XY*, et al.* OAS3 is a Co-Immune Biomarker Associated With Tumour Microenvironment, Disease Staging, Prognosis, and Treatment Response in Multiple Cancer Types. *Front Cell Dev Biol* **10**, 815480 (2022).

12. Xu N*, et al.* Identification of key DNA methylation-driven genes in prostate adenocarcinoma: an integrative analysis of TCGA methylation data. *J Transl Med* **17**, 311 (2019).

13. Rose AM. Cancer and the junkyard chromosome: how repeat DNA sequence on chromosome 19 influences risk of malignant disease. *Oncotarget* **9**, 31942-31944 (2018).

14. Biswas D, *et al.* Inhibiting BCKDK in triple negative breast cancer suppresses protein translation, impairs mitochondrial function, and potentiates doxorubicin cytotoxicity. *Cell Death Discovery* **7**, 241 (2021).

15. Sun T, Bi F, Liu Z, Yang Q. SLC7A2 serves as a potential biomarker and therapeutic target for ovarian cancer. *Aging (Albany NY)* **12**, 13281-13296 (2020).

16. Jiang S, *et al.* Lower SLC7A2 expression is associated with enhanced multidrug resistance, less immune infiltrates and worse prognosis of NSCLC. *Cell Communication and Signaling* **21**, 9 (2023).

17. Alessandrini F, Pezzè L, Menendez D, Resnick MA, Ciribilli Y. ETV7-Mediated DNAJC15 Repression Leads to Doxorubicin Resistance in Breast Cancer Cells. *Neoplasia* **20**, 857-870 (2018).

18. Zhang H, *et al.* Onco-miR-24 regulates cell growth and apoptosis by targeting BCL2L11 in gastric cancer. *Protein Cell* **7**, 141-151 (2016).

19. Tarsounas M, Sung P. The antitumorigenic roles of BRCA1–BARD1 in DNA repair and replication. *Nature Reviews Molecular Cell Biology* **21**, 284-299 (2020).

20. Feng Z, *et al.* Could CTSK and COL4A2 be specific biomarkers of poor prognosis for patients with gastric cancer in Asia?—a microarray analysis based on regional population. *Journal of Gastrointestinal Oncology* **11**, 386-401 (2020).

21. JingSong H, *et al.* siRNA-mediated suppression of collagen type iv alpha 2 (COL4A2) mRNA inhibits triple-negative breast cancer cell proliferation and migration. *Oncotarget* **8**, 2585-2593 (2017).

22. Zhuang W, Zhang C, Hao F, Sun X. Baculoviral IAP Repeat Containing 6 (BIRC6) Is a Predictor of Prognosis in Prostate Cancer. *Med Sci Monit* **24**, 839-845 (2018).

23. Hao T, Wang Z, Yang J, Zhang Y, Shang Y, Sun J. MALAT1 knockdown inhibits prostate cancer progression by regulating miR-140/BIRC6 axis. *Biomed Pharmacother* **123**, 109666 (2020).

24. Dong X, *et al.* Elevated expression of BIRC6 protein in non-small-cell lung cancers is associated with cancer recurrence and chemoresistance. *J Thorac Oncol* **8**, 161-170 (2013).

25. Gómez Bergna SM, *et al.* Exploring the Role of the Inhibitor of Apoptosis BIRC6 in Breast Cancer: A Database Analysis. *JCO Clinical Cancer Informatics*, e2200093 (2022).

26. Lin R, *et al.* Suppression of latent transforming growth factor-β (TGF-β)-binding protein 1 (LTBP1) inhibits natural killer/ T cell lymphoma progression by inactivating the TGF-β/Smad and p38MAPK pathways. *Experimental Cell Research* **407**, 112790 (2021).

27. Saraon P*, et al.* Proteomic Profiling of Androgen-independent Prostate Cancer Cell Lines Reveals a Role for Protein S during the Development of High Grade and Castration-resistant Prostate Cancer. *Journal of Biological Chemistry* **287**, 34019-34031 (2012).

28. Cai R*, et al.* LTBP1 promotes esophageal squamous cell carcinoma progression through epithelial-mesenchymal transition and cancer-associated fibroblasts transformation. *Journal of Translational Medicine* **18**, 139 (2020).

29. Zhang J, Deng H, Wang J. LTBP1 promotes the progression of triple negative breast cancer via activating the RhoA/ROCK signaling pathway. *Cancer Insight* **3**, (2023).

30. Gu H*, et al.* Immune suppressive signaling regulated by latent transforming growth factor beta binding protein 1 promotes metastasis in cervical cancer. *Braz J Med Biol Res* **55**, e12206 (2023).

31. Groenendyk J*, et al.* Loss of the fructose transporter SLC2A5 inhibits cancer cell migration. *Front Cell Dev Biol* **10**, 896297 (2022).

32. Nourmohammadi Abadchi S*, et al.* MLH1 Loss in Primary Prostate Cancer. *JCO Precis Oncol* **7**, e2200611 (2023).

33. Bonadona V*, et al.* Cancer Risks Associated With Germline Mutations in MLH1, MSH2, and MSH6 Genes in Lynch Syndrome. *JAMA* **305**, 2304-2310 (2011).

34. Abida W*, et al.* Genomic correlates of clinical outcome in advanced prostate cancer. *Proceedings of the National Academy of Sciences* **116**, 11428-11436 (2019).

35. Sowalsky AG*, et al.* Loss of Wave1 gene defines a subtype of lethal prostate cancer. *Oncotarget; Vol 6, No 14*, (2015).

36. Cai H*, et al.* In Vivo Application of CRISPR/Cas9 Revealed Implication of Foxa1 and Foxp1 in Prostate Cancer Proliferation and Epithelial Plasticity (2022).

37. Takayama K*, et al.* Integrative analysis of FOXP1 function reveals a tumor-suppressive effect in prostate cancer. *Mol Endocrinol* **28**, 2012-2024 (2014).

38. Yuan G*, et al.* Elevated NSD3 histone methylation activity drives squamous cell lung cancer. *Nature* **590**, 504-508 (2021).

39. Nuñez Y, Vera S, Baeza V, Gonzalez-Pecchi V. NSD3 in Cancer: Unraveling Methyltransferase-Dependent and Isoform-Specific Functions. *Int J Mol Sci* **25**, (2024).

40. Bakht MK*, et al.* Identification of alternative protein targets of glutamate-ureido-lysine associated with PSMA tracer uptake in prostate cancer cells. *Proceedings of the National Academy of Sciences* **119**, e2025710119 (2022).

41. Di Y*, et al.* The c-MYC-WDR43 signalling axis promotes chemoresistance and tumour growth in colorectal cancer by inhibiting p53 activity. *Drug Resistance Updates* **66**, 100909 (2023).

42. Sun H*, et al.* WD repeat domain 43 promotes malignant progression of non-small cell lung cancer by regulating CDK2. *The International Journal of Biochemistry & Cell Biology* **151**, 106293 (2022).

43. Li Z*, et al.* WD40 repeat 43 mediates cell survival, proliferation, migration and invasion via vimentin in colorectal cancer. *Cancer Cell International* **21**, 418 (2021).

44. Ruan Y, Xu H, Ji X. High expression of NPM1 via the Wnt/β-catenin signalling pathway might predict poor prognosis for patients with prostate adenocarcinoma. *Clin Exp Pharmacol Physiol* **49**, 525-535 (2022).

45. Loubeau G*, et al.* NPM1 silencing reduces tumour growth and MAPK signalling in prostate cancer cells. *PLoS One* **9**, e96293 (2014).

46. Liu XS*, et al.* NPM1 Is a Prognostic Biomarker Involved in Immune Infiltration of Lung Adenocarcinoma and Associated With m6A Modification and Glycolysis. *Front Immunol* **12**, 724741 (2021).

47. Gordon CA, Gong X, Ganesh D, Brooks JD. NUSAP1 promotes invasion and metastasis of prostate cancer. *Oncotarget* **8**, 29935-29950 (2017).

48. Liang B, Wang J. EVI1 in Leukemia and Solid Tumors. *Cancers (Basel)* **12**, (2020).

49. Ma Y*, et al.* CRISPR-mediated MECOM depletion retards tumor growth by reducing cancer stem cell properties in lung squamous cell carcinoma. *Molecular Therapy* **30**, 3341-3357 (2022).

50. Han L*, et al.* Comprehensive characterization of PKHD1 mutation in human colon cancer. *Cancer Med* **13**, (2024).

51. Ward CJ*, et al.* Germline PKHD1 mutations are protective against colorectal cancer. *Human Genetics* **129**, 345-349 (2011).

52. Huang J*, et al.* The Role of CTNNA1 in Malignancies: An Updated Review. *J Cancer* **14**, 219-230 (2023).

53. Josson S, Chung LW, Gururajan M. microRNAs and Prostate Cancer. *Adv Exp Med Biol* **889**, 105-118 (2015).

54. Berthon AS, Szarek E, Stratakis CA. PRKACA: the catalytic subunit of protein kinase A and adrenocortical tumors. *Front Cell Dev Biol* **3**, 26 (2015).

55. Angrisani A, Di Fiore A, De Smaele E, Moretti M. The emerging role of the KCTD proteins in cancer. *Cell Communication and Signaling* **19**, 56 (2021).

56. Qiu X*, et al.* Identification of m6A-Associated Gene DST as a Prognostic and Immune-Associated Biomarker in Breast Cancer Patients. *Int J Gen Med* **15**, 523-534 (2022).

57. Huang R*, et al.* Co-expression Analysis of Genes and Tumor-Infiltrating Immune Cells in Metastatic Uterine Carcinosarcoma. *Reproductive Sciences* **28**, 2685-2698 (2021).
