# Supplementary material for: Rare pathogenic structural variants show potential to enhance prostate cancer germline testing for African men
Source: Nat Commun. 2025 Mar 10;16:2400. doi: 10.1038/s41467-025-57312-9 (PMC11893795; doi:10.1038/s41467-025-57312-9)
Supplement: Supplementary file 3 — Description of Additional Supplementary Files [file 41467_2025_57312_MOESM3_ESM.pdf]

Description of Additional Supplementary Files

Supplementary Data 1: The detail of all dbVar studies (dbVar study name and ID) and reported allele frequencies of potentially pathogenic SVs identified in this study.

Supplementary Data 2: Rare cancer-related potentially pathogenic SV candidates identified in this study.

Supplementary Data 3: Detailed TITAN analysis used to predict somatic loss of heterozygosity (LOH) for potentially pathogenic SV candidates identified in this study.
